# Long-term ecological research in southern Brazil grasslands: Effects of grazing exclusion and deferred grazing on plant and arthropod communities

**DOI:** 10.1371/journal.pone.0227706

**Published:** 2020-01-13

**Authors:** Pedro M. A. Ferreira, Bianca O. Andrade, Luciana R. Podgaiski, Amanda C. Dias, Valério D. Pillar, Gerhard E. Overbeck, Milton de S. Mendonça, Ilsi I. Boldrini

**Affiliations:** 1 Programa de Pós-Graduação em Ecologia e Evolução da Biodiversidade, Pontifícia Universidade Católica do Rio Grande do Sul, Porto Alegre, Rio Grande do Sul, Brazil; 2 Departamento de Botânica, Universidade Federal do Rio Grande do Sul, Porto Alegre, Rio Grande do Sul, Brazil; 3 Departamento de Ecologia, Universidade Federal do Rio Grande do Sul, Porto Alegre, Rio Grande do Sul, Brazil; 4 Programa de Pós-Graduação em Biologia Animal, Universidade Federal do Rio Grande do Sul, Porto Alegre, Rio Grande do Sul, Brazil; 5 Department of Agronomy and Horticulture, University of Nebraska, Lincoln, Nebraska, United States of America; Indiana State University, UNITED STATES

## Abstract

Grazing exclusion may lead to biodiversity loss and homogenization of naturally heterogeneous and species-rich grassland ecosystems, and these effects may cascade to higher trophic levels and ecosystem properties. Although grazing exclusion has been studied elsewhere, the consequences of alleviating the disturbance regime in grassland ecosystems remain unclear. In this paper, we present results of the first five years of an experiment in native grasslands of southern Brazil. Using a randomized block experimental design, we examined the effects of three grazing treatments on plant and arthropod communities: (i) deferred grazing (i.e., intermittent grazing), (ii) grazing exclusion and (iii) a control under traditional continuous grazing, which were applied to 70 x 70 m experimental plots, in six regionally distributed blocks. We evaluated plant community responses regarding taxonomic and functional diversity (life-forms) in separate spatial components: alpha (1 x 1 m subplots), beta, and gamma (70 x 70 m plots), as well as the cascading effects on arthropod high-taxa. By estimating effect sizes (treatments vs. control) by bootstrap resampling, both deferred grazing and grazing exclusion mostly increased vegetation height, plant biomass and standing dead biomass. The effect of grazing exclusion on plant taxonomic diversity was negative. Conversely, deferred grazing increased plant taxonomic diversity, but both treatments reduced plant functional diversity. Reduced grazing pressure in both treatments promoted the break of dominance by prostrate species, followed by fast homogenization of vegetation structure towards dominance of ligneous and erect species. These changes in the plant community led to increases in high-taxa richness and abundance of vegetation-dwelling arthropod groups under both treatments, but had no detectable effects on epigeic arthropods. Our results indicate that decision-making regarding the conservation of southern Brazil grasslands should include both intensive and alleviated levels of grazing management, but not complete grazing exclusion, to maximize conservation results when considering plant and arthropod communities.

## Introduction

Grasslands, when under productive climatic conditions, are disturbance-prone ecosystems strongly shaped by fire and grazing regimes [1–3]. Disturbance can be defined as ‘any event in time that disrupts ecosystem, community, or population structure, and changes resource pools, substrate availability, or the physical environment’ [4] or, more simply, as ‘any event partially or totally destroying plant biomass’ [5]. Either way, disturbance plays a key role on grassland species composition, diversity patterns on multiple scales, and ecosystem functioning [1,2,6–9].

Large grazing animals can be very selective as to what they forage [10]. In productive natural grasslands, where high-quality palatable plants can be found, large herbivores preferably graze these plants, avoiding patches dominated by less palatable taxa [11–13]. This preferential grazing promotes heterogeneity by creating a mosaic of patches under different grazing pressures in the landscape, with the selection of plants that share traits compatible with each local disturbance [7,8]. Conversely, grazing exclusion, or very low grazing intensities, may benefit species with certain traits, such as the tussock habit of many C4 grasses (e.g., [14,15]), and ligneous species such as shrubs [16]. In the absence of disturbance, such species may dominate, homogenize, and ultimately change the structure of large patches via competitive exclusion [17,18], and these effects may be detected in different spatial components of diversity [12,19]. In productive systems such as a large portion of South America grasslands (specifically, the Río de la Plata grasslands [20]), grazing actually maintains the levels of biodiversity indicators (in comparison with the absence or severe reduction of grazing), for example plant species richness [18] and ant species richness [21].

To evaluate disturbance-driven grassland heterogeneity, plant species richness and composition alone may not be the best descriptors, but the usefulness of a functional approach is well established [7]. Based on recurrent findings of correlated plant traits, it has been suggested that reduced sets of traits such as life forms may be good descriptors and predictors of ecosystem functioning under disturbance [22] or climate change [23].

The direct effects of grassland management (i.e., human-driven modifications of the grassland habitat, related to disturbances such as fire and grazing) on plant diversity and structure usually lead to secondary effects on other trophic levels of the ecosystem. Arthropod faunal communities are in general very responsive to the presence and abundance of large herbivores (see a review in [24]), being mostly influenced by changes in resource availability (e.g., plant quality and quantity), habitat complexity (e.g., plant height and heterogeneity of vertical structure) and abiotic conditions (e.g., temperature, moisture). Because arthropods represent a very diverse group in terms of life-history traits, and use different strata of the grassland habitat, their responses to grazing are highly variable, depending on the sensitivity and requirements of each taxa, and on grazer and vegetation type [25,26]. Associated to shifts on arthropod communities, several related ecosystem functions can be substantially affected by grassland management, such as food provisioning to other trophic levels, contribution to soil fertility, biological control and pollination [27].

The relationships between grazing, biological community dynamics and ecosystem processes are still poorly studied in subtropical ecosystems in southern Brazil, especially considering long-term monitoring. Grasslands in this region, locally known as *Campos Sulinos*, are relict ecosystems from drier and cooler periods that are stabilized until today by the action of large herbivores and fire [28,29]. There is evidence of the presence of large grazing herbivores in South American grasslands since the early Miocene [30,31]. After their extinction, grazing by domestic cattle has become widespread since the seventeenth century, and today cattle breeding is one of the most important economic activities in the region [32]. In fact, cattle breeding farms that use grasslands as natural forage sources encompass most of the grassland remnants in the region, since only 0.33% of southern Brazil grasslands are inserted in protected areas, and in most of them, the disturbance regime with cattle grazing and fire is absent [33]. However, the degree of resistance/resilience of these systems to grazing remains unclear, as do the consequences of interrupting or alleviating the disturbance regime [34]. Long-term ecological research (LTER) is essential to address these questions. Although LTER has greatly improved our understanding of ecosystem dynamics (reviews in [35,36]), very little of this evidence comes from the Southern Hemisphere, and even less from grasslands in southern South America (but see [18,37–39]).

In this paper, we present results of the first five years of an experiment established since 2010 in native grasslands at six LTER sites in southern Brazil grasslands. Using a randomized block experimental design, we examined the effects of two treatments (i.e., grazing management options) on plant and arthropod communities: (i) deferred grazing, and (ii) grazing exclusion. To do so, we also established in each block a control treatment under traditional continuous grazing. First, we hypothesized that management exclusion will lead to an overall decrease in grassland plant species richness, diversity and functional diversity (as seen after fire disturbance in similar ecosystems; e.g., [17]). To test this, we evaluated plant community responses in separate spatial components using a framework for diversity partitioning. Then, we expected that both grazing exclusion and deferred grazing would lead to shifts in the dominance structure among plant life forms, and ultimately in habitat structure properties. Finally, we explored secondary effects of grazing exclusion and deferred grazing on the community of arthropods [40], as represented by major arthropod taxa both from soil surface and vegetation strata, with different requirements and influences on ecosystem processes. Our study provides novel empirical evidence to support management strategies for southern Brazil grasslands, especially concerning the effects of grazing exclusion or reduced grazing intensity.

## Material and methods

### Sampling sites

**Fig 1:**
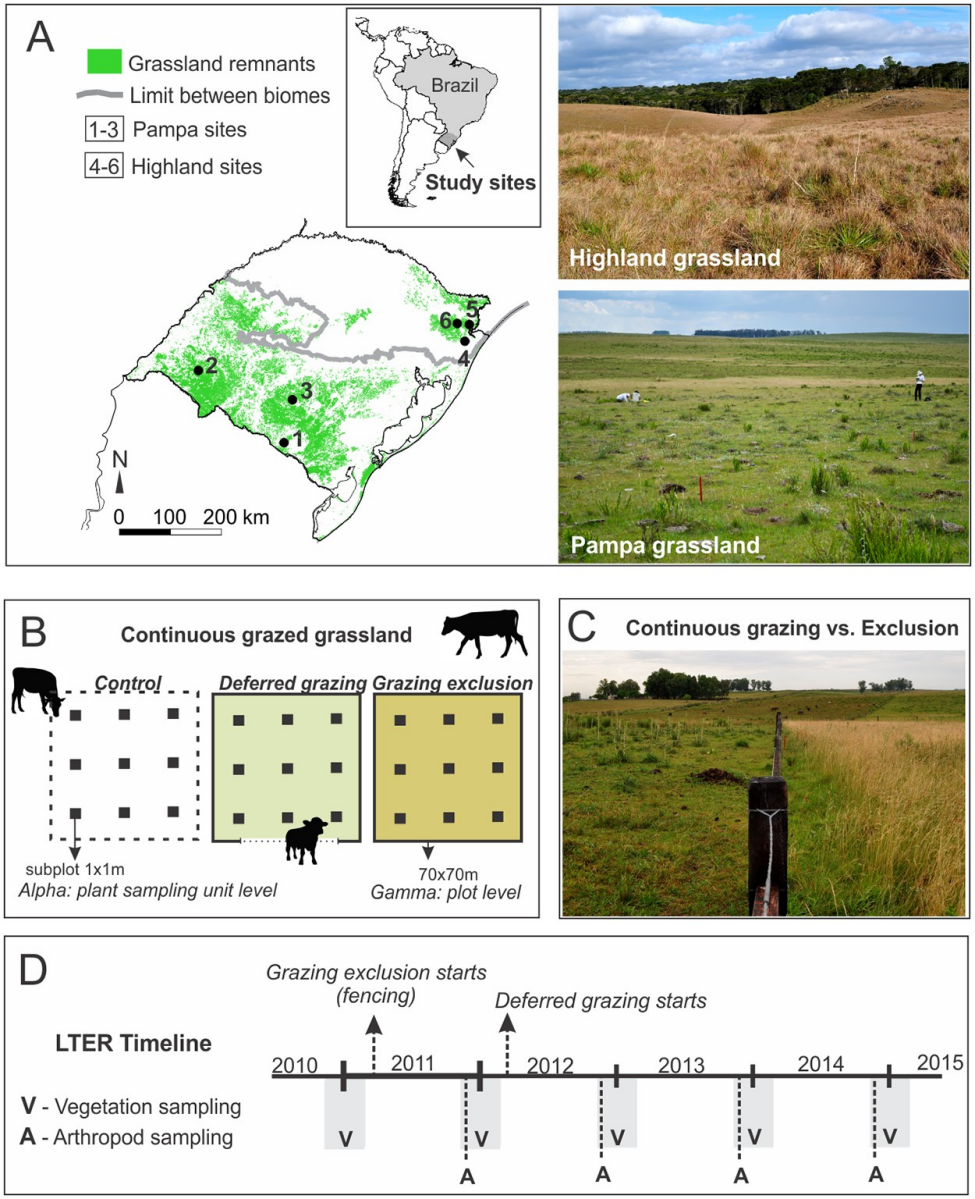
Study sites and sampling design. A. Study site locations and grassland remnants in southern Brazil; pictures with typical landscapes in Pampa and highland grasslands. Study sites: 1- Aceguá, 2- Alegrete, 3- Lavras do Sul, 4- Aratinga Ecological Station, 5- Aparados da Serra National Park, 6- Tainhas State Park. B. Block with three experimental plots, each randomly subjected to one treatment: ‘control’, under continuous cattle access from the larger, enclosing grazing area where the block is located, ‘deferred grazing’, with controlled cattle access, and complete ‘grazing exclusion’; the subsampling design for description of plant communities is indicated within each experimental plot. C. a picture is showing contrasting vegetation structure after one year of exclusion (right side of the fence) compared to the grazed control (left side of the fence). D. Timeline showing sampling events and starting points of treatments.

Our study area comprised six sites in Southern Brazil (Fig 1A; S1 Table). The *Campos Sulinos* span across two different phytogeographic domains: Pampa and Atlantic Forest. Atlantic forest grasslands occur in the highlands of southern Brazil (hereafter ‘highland grasslands’, as in [41]). We selected three sites in each domain. Pampa grasslands cover large continuous areas, and forests are mostly restricted to riverbeds and specific edaphic conditions. Highland grasslands shape mosaics with forests in the landscape [42,43]. Evindence suggests that forests in the region have been expanding over grassland vegetation, and that this expansion is mediated by disturbances such as fire and grazing [44–46]. Pampa sites were located at Aceguá, Alegrete and Lavras do Sul municipalities. Highland sites were at the Aparados da Serra National Park (Cambará do Sul municipality), Aratinga Ecological Station, and Tainhas State Park (the last two in São Francisco de Paula municipality). Grasslands at all sites have been under continuous cattle grazing, arguably since the introduction of domestic cattle in the 17^th^ century in the Pampa and 18^th^ century in the highlands. No fire event took place in any of the sites during the experiment.

### Experimental design

This study is part of a Long-Term Ecological Research study (LTER/PELD *Campos Sulinos*–CNPq). Here we report results obtained during the first five years (2010–2014). The experiment consisted of a randomized block design (Fig 1B). At each site, we delimited three plots of 70 x 70 m, which were randomly assigned to one of three treatments of grazing management regimes using cattle: (i) deferred grazing (i.e., intermittent cattle access for grazing), (ii) grazing exclusion, and (iii) traditional continuous grazing (control).

In each block (Fig 1B), the plot under traditional continuous grazing was not fenced and thus was freely accessible to grazing cattle from the enclosing grassland area, under a stocking rate commonly applied by the manager at each site (0.6 to 0.9 animal units per hectare on average, with variations depending on the season, but held roughly constant within the same site). The plot under deferred grazing was fenced, with a gate to control animal access, and the treatment consisted of grazing events with grazing pressure concentrated in a short period of time (1–2 days), while during the interval between grazing events the plot remained excluded (deferred) from grazing. The interval between grazing events was the accumulated thermal sum of 700 degrees-day (sum of daily mean temperatures larger than zero °C). The stocking rate (kg of animal live weight) used in each grazing event was adjusted to obtain a post-grazing aboveground residual of approximately 1,500 kg of dry plant biomass per hectare. The adjustment was based on the available aboveground biomass estimated by the comparative yield method [47]. The interval between grazing events was variable depending on site and season, and ranged between 22 and 37 days. These procedures aimed to maintain forage availability at optimal levels, considering perennial C4 grasses [48], to promote habitat heterogeneity and less accumulation of dead biomass, and ultimately to enhance ecosystem resilience [49]. It is important to mention that the absolute number of grazing animals used in the deferred plots during the grazing events was usually higher than the number of animals in the traditional grazing plots in a similar time window, which ultimately results in higher grazing pressure in that short period of time. However, the overall grazing pressure was lower in deferred plots because of the time they were excluded from grazing. Finally, grazing exclusion consisted on fenced paddocks that completely precluded the entry of grazing animals.

Delimitation of experimental plots and construction of fences for deferred grazing and grazing exclusion took place in southern hemisphere spring/summer of 2010–2011. As soon as fences were placed, there was no access of cattle into both deferred and exclusion plots, which promoted initial plant biomass accumulation. The deferred grazing management started in late 2011 to early 2012. We report our results referring to 2010 as ‘year zero’ of the experiment, and the following years as years one to four. See Fig 1D for a timeline with sampling events and management starting points.

### Plant community and habitat structure sampling

We sampled grassland vegetation in all experimental plots during southern hemisphere spring/summer starting in 2010 before the fencing. We conducted this first sampling event (before starting the experiment) to evaluate if plant communities were uniform between experimental plots. This was confirmed for most descriptors of plant taxonomic and functional diversity compared to the control, with the exception of therophytes in deferred grazing plots, and tussocks in grazing exclusion plots. We repeated vegetation description of all plots during the same period starting in 2011, 2012, 2013, and 2014 (Fig 1D). In each experimental plot, we sampled vegetation using nine 1 x 1 m subplots (systematically located in a 3 x 3 grid with 17 m between units; Fig 1B), which were permanently marked. In each subplot, we surveyed all plant species that were present and estimated their cover using the decimal scale of Londo [50]. We also estimated vegetation height in five points per subplot, as well as the percentage of bare soil, dung, and standing dead biomass. Finally, we collected plant biomass in six 25 x 25 cm subplots contiguous to vegetation sampling subplots to measure dry plant biomass (only in the last three monitoring years).

**Fig 2:**
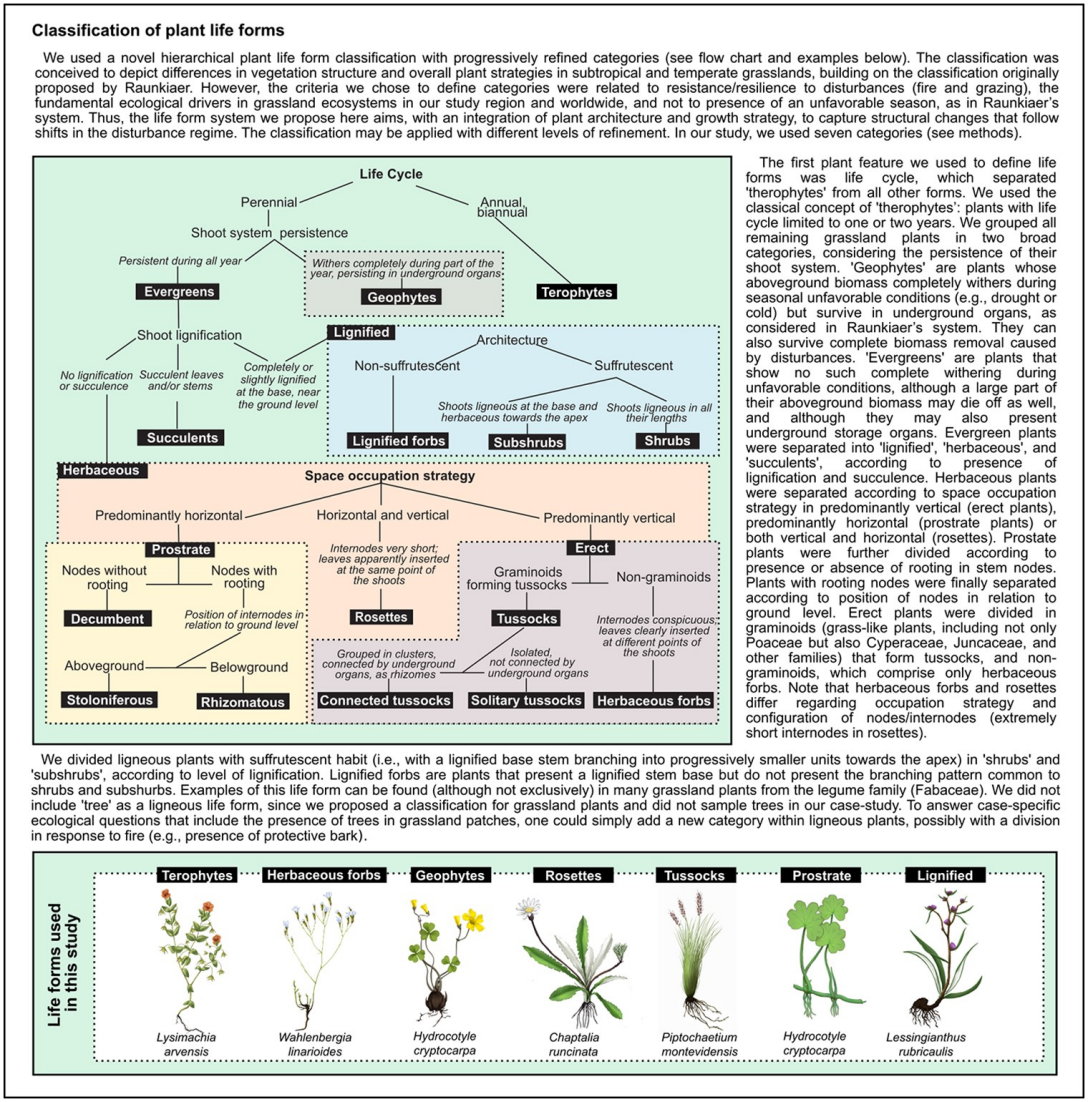
Classification of grassland plant life forms.

We organized the plant community data in a matrix containing average cover values of species describing sampling units (1 x 1 m subplots or 70 x 70 m plots) in each of the five monitoring years (matrix **W**_**P**_). We derived different versions of matrix **W**_**P**_ containing information per treatments, years and/or grain (subplot or plot) separately, depending on the analysis. We used plant life forms as binary functional traits, in order to estimate plant functional diversity. We classified plant species into seven life form categories: terophytes, geophytes, herbaceous forbs, tussocks (both connected and solitary), rosettes, lignified (including lignified forbs, shrubs, and subshrubs), and prostrate (including decumbent, stoloniferous, and rhizomatous plants). See Fig 2 for explanation of each life form category and S2 Table for a list of all sampled species with their respective life form category. In this classification, life forms were based on features such as habit, architecture, level of lignification and strategy of horizontal occupation, which are traits responsive to shifts in management, and good descriptors of vegetation structure. Life forms, which are nominal traits, were expanded into binary traits organized in a matrix **B** of species described by life form categories. To evaluate changes in dominance of life forms across the years of sampling and under different grazing treatments, we generated a matrix **T** with community weighted mean (CWM) traits using matrix multiplication **T** = **W**_**P**_**B** [51]. Actually, CWMs in this case are proportions of life form categories in each sampling unit. Note that we combined some ‘terminal’ life forms presented in Fig 2 into coarser categories, in order to capture the collective response of important groups that shape grassland structure and landscape, such as lignified and prostrate plants, to the different grazing treatments.

### Arthropod sampling

We sampled arthropod communities in all plots during Summer (November/December) from 2011 to 2014, except for one site in which arthropod sampling could not be carried out in 2011. Epigeic arthropods were sampled with pitfall traps, and arthropods from the grassland vegetation with sweeping net. At each experimental plot, eight pitfall traps were installed at least 15 m apart. The trap consisted in a 500 mL transparent plastic jar (10 cm diameter, 12 cm depth) filled with 150 mL of formalin (3% formaldehyde), which remained open for seven days. To reduce the evaporation rate of formalin, and to protect the traps from direct rainfall, green plastic dishes sustained by wooden sticks were used as rain guards. Arthropods were gathered from vegetation with a sweep net (50 cm wide; sampling area of 0.1 m^2^) along four parallel transects in each experimental plot, being all individuals pooled together in a single sample. Vegetation was swept twice every year: before pitfall installation and before removal. See S1 Fig for images of the sampling process. Both samples were also pooled in a single sample per plot. Collected specimens were stored in a plastic bag with ethyl acetate. All individuals were preserved in ethanol 80%, counted and sorted in major taxonomic groups (e.g., orders), and stored in the Laboratório de Ecologia de Interações (LEIN) at Universidade Federal do Rio Grande do Sul. Arthropod community data were summarized in matrix **W**_**A**_, of plots described by the number of individuals of the most abundant taxa in both strata.

### Data analysis

We adopted an effect size approach by estimating bootstrap confidence intervals [52,53] for standardized effect sizes contrasting treatments vs. control on plant and arthropod communities and habitat structure. We estimated these effects considering plant species composition and their relative abundances, species richness and diversity, proportions of plant life forms, and habitat descriptors. For plant species, we also partitioned taxonomic and functional diversity into different spatial components (see details below). For arthropod communities, we considered overall high-taxa richness (order level) and total abundance, and the abundance of each of the dominant orders.

We used the plot level (70 x 70 m) in all analyses that aimed to estimate the effect of deferred grazing and grazing exclusion on community and habitat descriptors (subplot mean values for plant data, and totals per plot for arthropods). For the plant diversity partitioning analysis, we used data pooled to both spatial grains (1 x 1 m subplots and 70 x 70 m plots).

We partitioned plant taxonomic and functional diversity into different spatial components using the framework presented by [54]. Functional diversity (Rao entropy) was calculated using plant life form traits, which reflect different strategies of habitat occupancy and response to disturbances. Rao entropy was computed by using Gower dissimilarities [55] between species (based on life forms) for functional diversity, and by using a unity matrix with null diagonal for taxonomic diversity [54]. In our case, the within experimental plot averages of diversity in 1 x 1 m subplots represented the alpha component, and the diversity for the 70 x 70 m plots represented the gamma component. As suggested by [54], we used unweighted species relative abundances and applied Jost’s correction derived from equivalent numbers [56] for both taxonomic and functional partitioning, and for both alpha and gamma components, so that equivalent beta diversity was computed as equivalent gamma divided by equivalent alpha diversity.

We evaluated the effect of deferred grazing and grazing exclusion on grassland plant and arthropod communities by calculating effect sizes, which provide both the magnitude and precision of the effect estimation [57]. We measured the magnitude of the effect of deferred grazing and grazing exclusion on response variables for each sampling year with Hedges’ unbiased standardized effect size estimator [58]:

$$g=\frac{M1-M2}{s}J$$

where M1 is the mean value of a response variable in deferred or exclusion experimental plots, M2 is the mean value of the same variable in control plots, *s* is the pooled standard deviation across experimental plots, and J is a correction to reduce bias in small (n = 6 blocks) sampling sizes. Response variables consisted on plant taxonomic and functional diversity (alpha, beta and gamma components), plant life forms, habitat descriptors, arthropod high-taxa richness, total abundance, and abundance of each taxa. We calculated 95% bootstrap confidence intervals, with 10,000 iterations and the bias-corrected-and-accelerated (BCa) method, to test if observed effects of deferred grazing and grazing exclusion were different from what is expected by chance within the dataset (BootES R package; [59]). We controlled the block effect (sampling site) by subtracting mean values of each block from each observed value prior to effect size estimation [60]. We report only significant results in the text by addressing the effect of deferred grazing and grazing exclusion on a given variable as ‘positive’ or ‘negative’ when the confidence interval did not include zero.

Finally, to compare the results of plant taxonomic diversity (alpha and gamma components) between treatments and across years, we plotted diversity profiles using Renyi’s entropy values, which enables comparisons of diversity between datasets using multiple diversity indexes simultaneously [61,62].

## Results

### Biological communities

We sampled 441 plant species distributed in 49 families, and 21 macrofaunal taxonomic groups, representing a total of 30,169 epigeic arthropods, and 23,030 vegetation arthropods. Seven arthropod taxa were the most abundant in both strata (Araneae, Coleoptera, Hemiptera, Hymenoptera, Orthoptera, Thysanoptera) and were used in the analyses. See S2 Table for a list of all plant species and their respective life forms. In addition, see S3 Table for relative cover values of plant life forms in each site, year and treatment, and S4 and S5 Tables with total abundance of soil and vegetation arthropod orders in each site, year and treatment.

### Habitat structure

**Fig 3:**
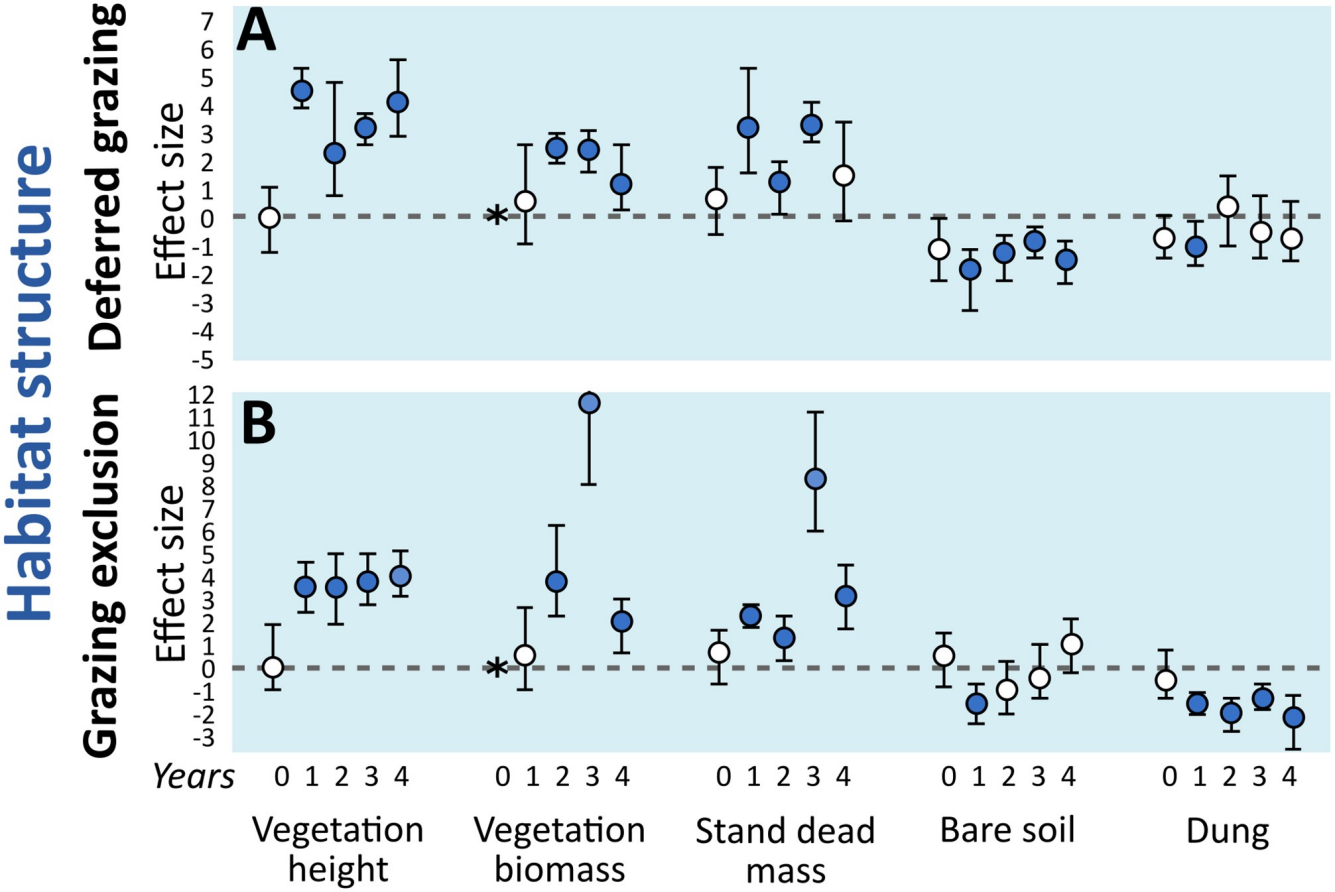
Mean effect size (Hedges’ g) of grazing exclusion and deferred grazing on grassland habitat descriptors. Numbers 0–4 correspond to years of treatment (2010–2014). Error bars are bootstrap 95% confidence intervals. Treatment effect is significantly positive or negative (filled circles) when intervals do not overlap with zero. Vegetation biomass was not sampled in the first year (*).

The effects of both deferred grazing and grazing exclusion on habitat structure indicators were mostly positive for vegetation height, plant biomass and standing dead biomass. Presence of dung was negatively affected by the exclusion, and unaffected by the deferred grazing. Percentage of bare soil was unaffected by grazing exclusion except for the first year of experiment, but negatively affected by deferred grazing management in all years (Fig 3).

### Plant life forms

**Fig 4:**
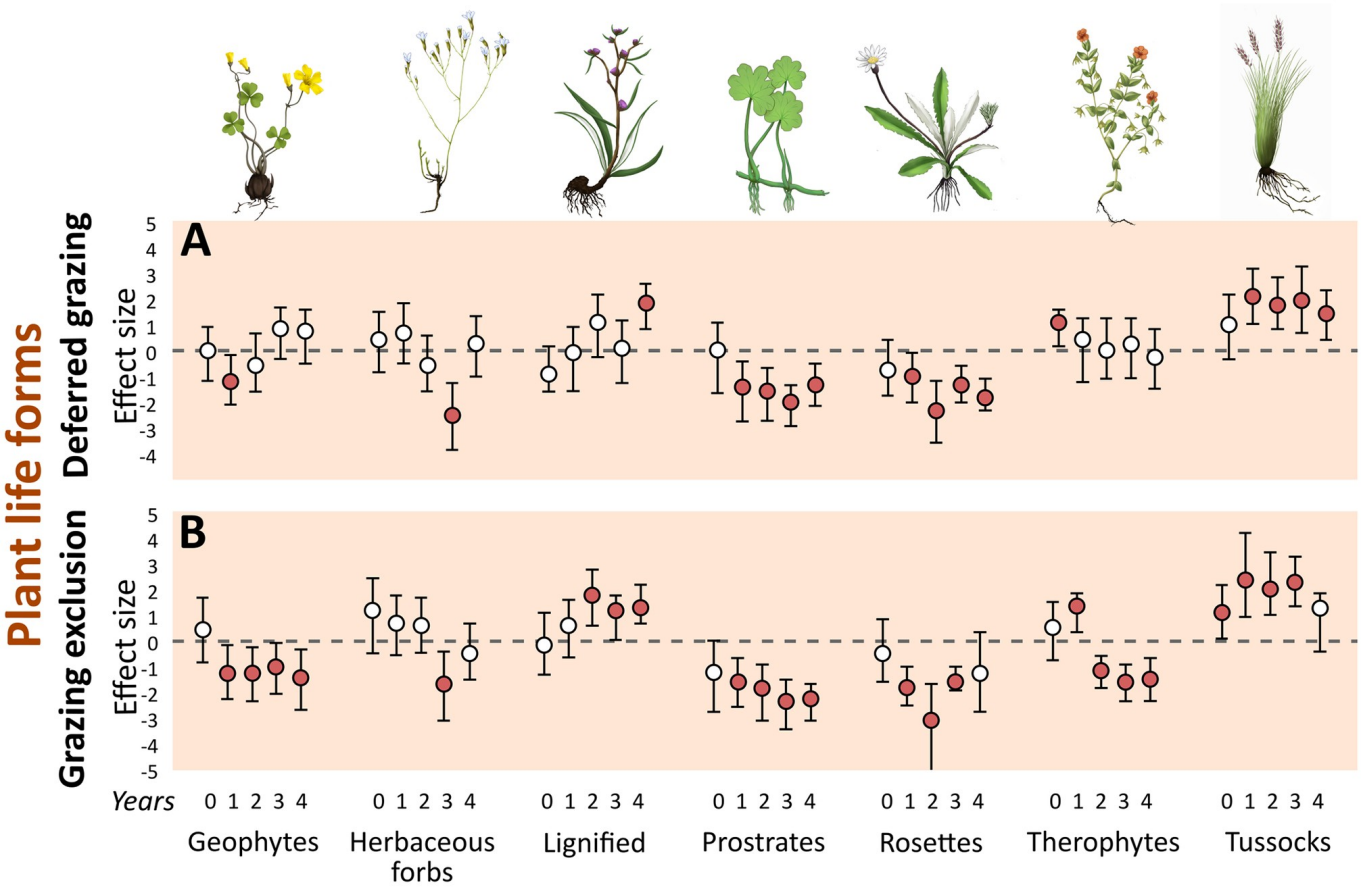
Mean effect size (Hedges’ g) of grazing exclusion and deferred grazing on grassland plant life forms. Effect based on standardized cover values. Numbers 0–4 correspond to years of treatment (2010–2014). Error bars are bootstrap 95% confidence intervals. Treatment effect is significantly positive or negative (filled circles) when intervals do not overlap with zero.

Regarding the relative contribution of plant life forms (community-weighted mean traits), the effect of grazing exclusion was positive for tussocks in the first three years of experiment and for lignified plants in the last three years (Fig 4). The effect of exclusion was also positive for therophytes in the first year of experiment, although it was negative in the following years. The effect of exclusion was negative for prostrate plants and geophytes in all years of experiment, and for herbaceous forbs in the third year and rosette plants in the first three years (Fig 4). The effect of deferred grazing on plant life forms was overall similar to the effect of grazing exclusion, with the exception of geophytes and therophytes (no observed effect). In addition, the positive effect of deferred grazing on lignified plants was detected only in the last year.

### Plant taxonomic and functional diversity

**Fig 5:**
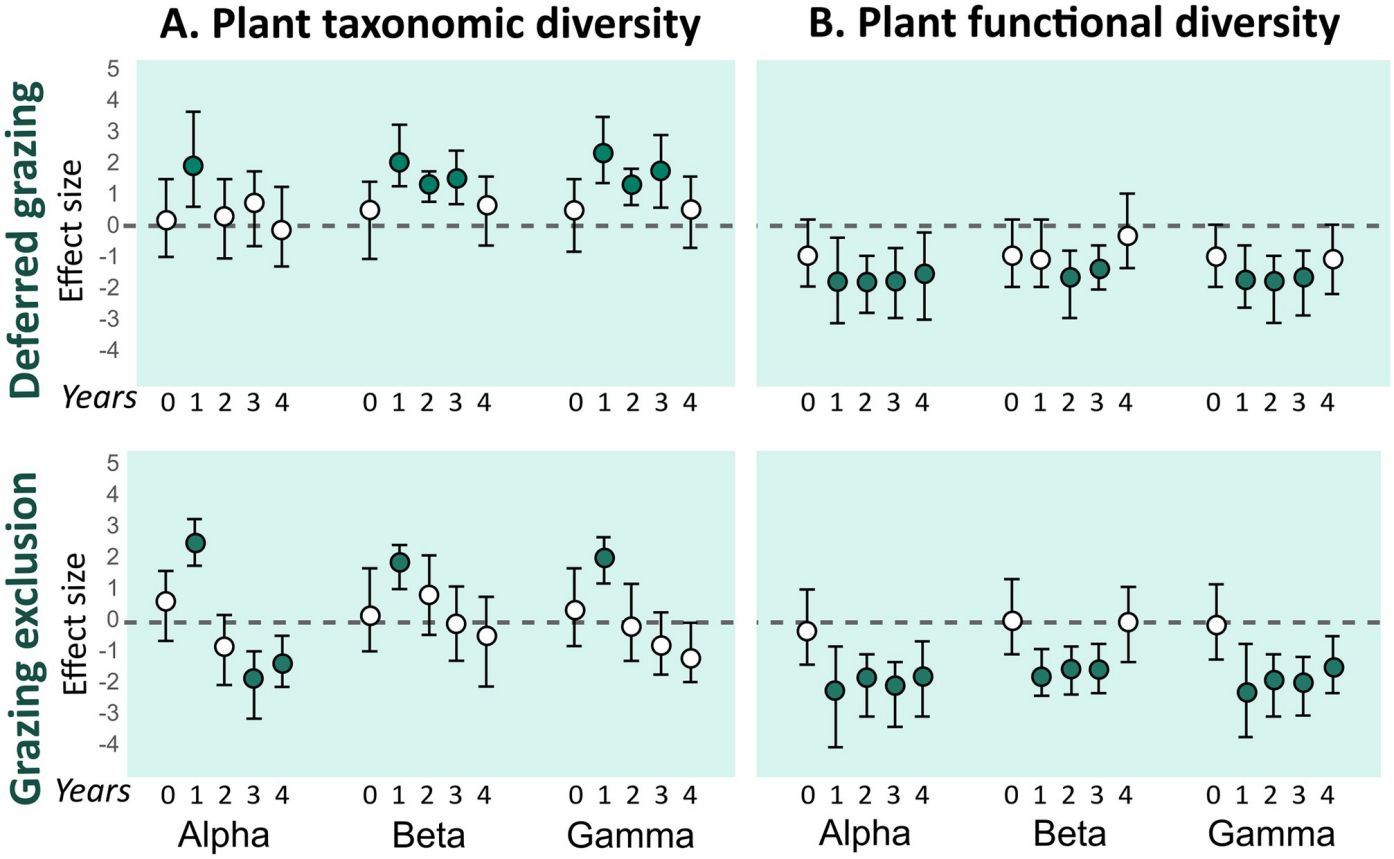
Mean effect size (Hedges’ g) of deferred grazing and grazing exclusion on alfa, beta, and gamma plant taxonomic and functional diversity. A. Taxonomic diversity. B. Functional diversity. Numbers 0–4 in the x-axis correspond to years of treatment (2010–2014). Error bars are bootstrap 95% confidence intervals. Treatment effect is significantly positive or negative (filled circles) when intervals do not overlap with zero.

Effects of grazing exclusion on plant taxonomic diversity were positive in the first year of experiment for all spatial components of diversity. After the first year, the effect becomes progressively lower for all components, up to the point of being significantly negative for the alpha component (last two years). For the beta and gamma components, the effect of grazing exclusion was not significant after the first years’ positive effect, but became numerically lower as the experiment progressed in time. Conversely, the effect of deferred grazing on plant taxonomic diversity was mostly positive: in the first year for the alpha component, and in the first three years for the beta and gamma components (Fig 5A).

Effects of grazing exclusion and deferred grazing on plant functional diversity were similar: negative for the alpha and gamma components in all years of experiment. The beta functional component was negatively affected by grazing exclusion in the first three years and by deferred grazing in the second and third years (Fig 5B).

**Fig 6:**
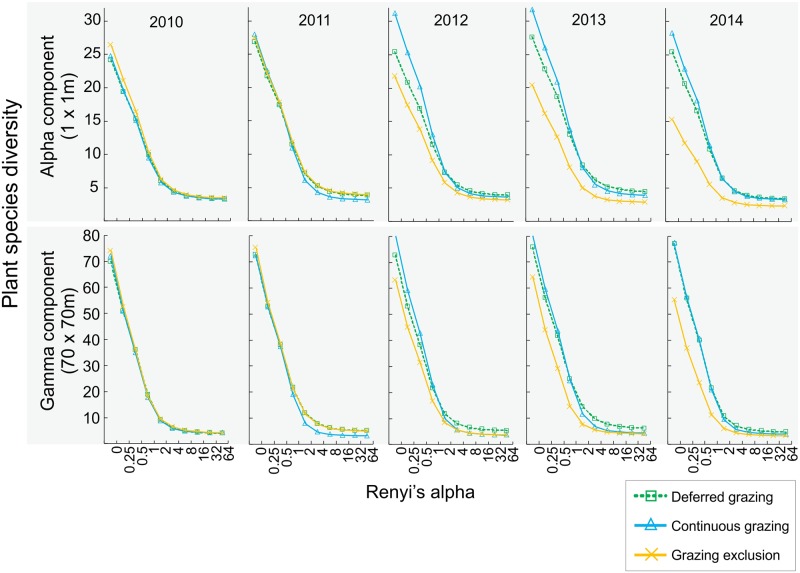
Plant species diversity profiles described by Renyi’s entropy values for each year of experiment. Data from six grassland sites separated by treatment (continuous grazing [solid blue lines], deferred grazing [dashed green lines], and grazing exclusion [solid orange lines]), and year (2010–2014). Data pooled according to spatial component (alpha [1 x 1 m subplots, n = 162] and gamma [70 x 70 m plots, n = 18]).

Diversity profiles using Renyi entropy values revealed similar plant species diversity (i.e., overlapping profiles) under continuous and deferred grazing in all years of experiment, and for both spatial components. Considering the alpha component, we detected lower plant diversity under grazing exclusion in the last three years of the experiment (Fig 6A). For the gamma component, we also detected lower diversity under grazing exclusion, but this was clearer in the last year of experiment (Fig 6B).

### Arthropods

**Fig 7:**
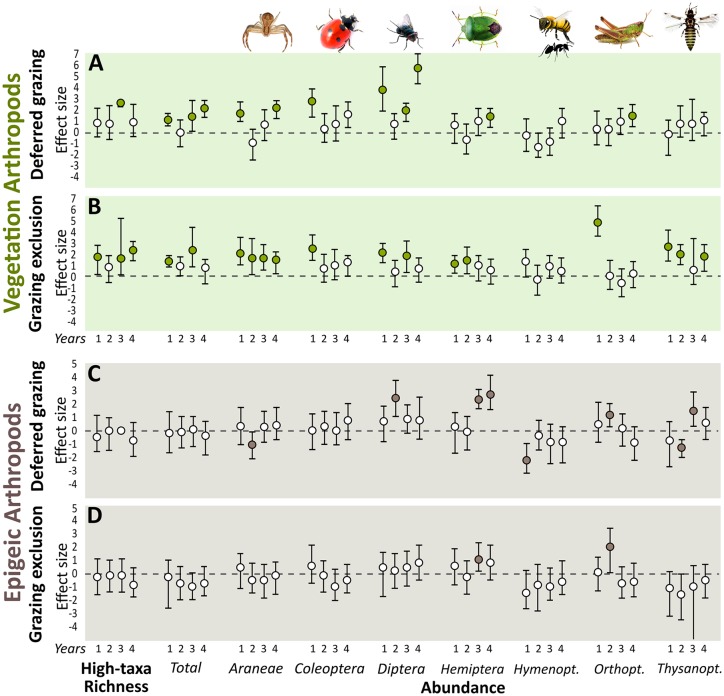
Mean effect size (Hedges’ g) of grazing exclusion and deferred grazing on soil and vegetation arthropods abundance. A. Vegetation arthropods. B. Epigeic arthropods. Numbers 1–4 correspond to years of treatment (2011–2014). Error bars are bootstrap 95% confidence intervals. Treatment effect is significantly positive or negative (filled circles) when intervals do not overlap with zero.

Deferred grazing and grazing exclusion were mostly positive for vegetation arthropod high-taxa richness and total abundance, and influenced the abundance of many arthropod orders, although the response patterns were year- and group-specific. Specifically, deferred grazing positively affected arthropod high-taxa richness in the third year of the experiment, and the total abundance in the first, third and fourth years. Grazing exclusion affected high-taxa richness in the first, third, and fourth years, and total abundance in the first and third years. Araneae abundance was positively affected by grazing exclusion in all experimental years, but only in the first and last years under deferred grazing. Coleoptera, Diptera, Hemiptera, Thysanoptera, and Orthoptera abundances were positively influenced in at least one year by either grazing exclusion or deferred grazing (Fig 7).

Epigeic arthropod high-taxa richness and total abundances were not affected by deferred grazing or grazing exclusion treatments (Fig 7). Araneae, Diptera, Hemiptera, Hymenoptera, Orthoptera and Thysanoptera were positively or negatively affected in at least one experimental year, without a clear response pattern.

## Discussion

Here we used a controlled randomized experiment, established at six sites of natural grassland ecosystems, to test for the effects on plant and arthropod communities of four years of deferred (i.e., intermittent) grazing and grazing exclusion contrasted to the control treatment under continuous grazing. We hypothesized that management exclusion would negatively impact plant diversity descriptors, change habitat structure and the relative contributions of plant life forms, and ultimately affect arthropod communities through shifts in the plant community.

With the exception of a short-term (first year after grazing exclusion) positive effect on plant taxonomic diversity, we observed an overall negative effect of grazing exclusion on plant taxonomic and functional diversity. Deferred grazing, in contrast, promoted an increase in plant taxonomic diversity, especially for the beta and gamma components. Grazing exclusion and deferred grazing promoted shifts in habitat structure mediated by differential responses of plant life forms. All these modifications led to an overall positive effect on the abundance and high-taxa richness of arthropods associated to vegetation strata, but had no clear effects on major epigeic arthropod groups. Below, we discuss in detail the response of each of these descriptors to grazing exclusion and the alleviated grazing pressure that our deferred treatment represents.

### Habitat structure

Habitat structure was strongly affected by grazing exclusion and deferred grazing. Average vegetation height, plant biomass and standing dead biomass increased under both treatments contrasted to the control treatment under continuous grazing. Grazing exclusion also promoted obvious reduction of dung cover in all years, whereas this effect was not observed under deferred grazing. Interestingly, the deferred grazing promoted a reduction on bare soil in all years, which was not observed in the exclusion treatment (i.e., the deferred grazing treatment increased overall plant cover, whereas grazing exclusion did not). The increase in aboveground biomass and plant height directly impacts microclimatic characteristics by limiting sunlight, changing soil humidity, and drainage [38,63], while also creating temperature buffering [24]. However, the dense vegetation may increase the availability of shelter and occupation area for associated arthropods [64], and alter the environmental filters that assembly these communities (e.g., [65]). Under increasing amounts of dead biomass and taller vegetation, many grassland plant species may be outcompeted due to shading, tend to first decrease in relative contribution, and eventually disappear [66,67], leading to open soil between tussocks.

### Plant life forms and functional plant diversity

Both deferred grazing and grazing exclusion promoted the replacement of prostrate and rosette species by tussocks. The break of dominance by prostrate taxa was followed by a positive effect on the contribution of lignified species. However, the effect of deferred grazing on lignified species was detected only in the last year of sampling, contrasting with the positive effect of grazing exclusion observed in all but the first year after the treatments started. In a global synthesis on plant trait responses to grazing, [7] reported that grazing favored short, prostrate plants over tall, erect plants. Shifts in vegetation structure followed by disturbance suppression are commonly reported for grassland ecosystems worldwide. For example, [38] reported an increase of ligneous species over a previously predominantly herbaceous vegetation after disturbance suppression. Encroachment with ligneous species such as shrubs and subshrubs reduces overall plant species richness in grasslands [16]. Moreover, C4 grasses are negatively affected by shrubs through reduction in radiation, and probably below-ground competition [68], which may push the herbaceous-dominated grasslands towards a woody-dominated system faster than expected. In our last year of sampling, grazing exclusion had no effect on tussocks. This could be related to the abovementioned negative effect of lignified species on tussock-forming C4 grasses, coupled with the expected reduction of growth rates in the absence of biomass removal [69,70]. It is important to mention that our results related to ligneous species comprise shrubs (e.g., *Bacharis dracunculifolia* and *Campomanesia aurea*), subshrubs (e.g., several *Baccharis* species), and ligneous forbs (e.g., species belonging to families Fabaceae and Asteraceae) (see Fig 2 and S2 Table). In addition, the most representative ligneous species were in fact subshurbs (ca. 68% of ligneous species cover across all sampling years, especially *Baccharis* spp.–*B*. *crispa*, *B*. *pentodonta*, and *B*. *coridifolia*), although the distinction between shrubs and subshrubs is not often applied. In productive grasslands, the cessation of grazing is usually followed by a shift of dominance from species adapted to grazing, with predominantly horizontal growth and fast resource-acquisition, to species with predominantly vertical growth strategy, which are also usually less palatable to grazers and better competitors [71–75]. The negative effect of grazing exclusion over C3 grasses (i.e., most prostrate grass species in our case) with low fiber content and high nutritional value) is likely to reduce even further the forage quality of the grassland, making it difficult to later re-introduce grazing after exclusion for longer periods. The set of species/traits that predominate under grazing and fire [3,6] grants the resilience to disturbance associated with ecosystems that have been recently addressed as ‘old-growth grasslands’ [76].

Our results indicate that a lower grazing pressure, here emulated by our deferred treatment, can promote similar effects of grazing exclusion in the relative contribution of plant life forms in comparison with continuous grazing, at least considering the ‘prostrate/herbaceous vs erect/ligneous’ dichotomy discussed above. However, grazing exclusion promoted negative effects on geophytes and therophytes, whereas deferred grazing did not. This indicates that, in the long run, both plant life forms would be preserved under lower grazing intensities, whereas they would probably be locally extinct or severely reduced under grazing exclusion. Several rare and extinction-threatened species are geophytes (e.g., [77]). Many species with C3 photosynthetic pathway present in the southern Brazilian grasslands add to the quality of the natural pasture in the unfavorable season. Therefore, the implications of the local extinction of such species under grazing exclusion affect not only conservation issues, but also can be detrimental to the potential for cattle breeding, one of the most important economic activities in the region and the main economically viable alternative to land conversion and the consequent habitat loss [33,78]. Although geophytes may in fact resist the effects of grazing exclusion by surviving underground in the bud bank [79], this survival may be restricted to the first few years of exclusion, since bud banks tend to decrease in the absence of disturbances [80].

### Plant taxonomic diversity

The short-term increase in plant taxonomic diversity seen for all spatial components in the first year for both treatments (Fig 5) was related to the reduction of dominance by prostrate species, which are highly adapted to grazing conditions, coupled with the increase in representativeness of taxa that are comparatively poor competitors under grazing (e.g., lignified plants and tussocks; Fig 4). Our results agree with recent findings showing that short-term grazing exclusion could increase plant species richness and productivity [14], and with results related to increases in plant species richness and diversity after fire disturbance (e.g., [17]). However, the effect of grazing exclusion on plant taxonomic diversity after the first year of exclusion was either non-significant or negative. These findings indicate that the increased diversity after the break of dominance by prostrate species was followed by a surprisingly fast (less than two years) homogenization of vegetation structure towards another state (i.e., dominated by ligneous and erect species, as opposed to the previous prostrate-dominated structure). Current evidence points out that southern Brazil grasslands are resilient enough to return to their species-rich state after years of overgrazing [14]. Similarly, after many years of management-exclusion, species-poor grasslands from the south Brazilian highlands showed positive responses (i.e., increased plant species richness and diversity) to mowing as a restoration technique [81].

After the first year, grazing exclusion had no effect on plant beta and gamma taxonomic diversity, and had a negative effect on alpha (third and fourth years) components (Fig 5A). The early detection of the negative effect in the alpha component in comparison with the gamma component was probably related to local extinctions of rare species at the subplot level (1 x 1 m), which may take more time to be detected at the plot level (70 x 70 m), and was not detected at all under deferred grazing. The effect size of grazing exclusion on taxonomic diversity for the beta and gamma components showed a decreasing trend after the first year of experiment, suggesting that stronger negative effects are likely to be detected as the experiment goes on. Conversely, after the diversity peak of the first year of experiment, the deferred grazing treatment maintained the positive effect over plant taxonomic diversity in the next two years for the beta and gamma components, and had no effect for the alpha component. Coupled with the results on habitat structure variables and plant life forms discussed above, these results on beta and gamma plant diversity indicate that the intermediate disturbance intensity of the deferred treatment (considering grazing exclusion and continuous grazing as the extremes) promoted increased heterogeneity in the grassland plant communities. Large herbivores play a key role on grassland structural and functional aspects [82,83]. Grazing under moderate levels promotes enhanced plant species richness [18], and plant species diversity is higher in grazed grasslands compared to exclosures [38] in Uruguayan grasslands (which are spatially and phytogeographically close to the south Brazilian *Campos Sulinos* grasslands). This pattern is due to the suppression of competitively dominant species via competitive release promoted by grazing [18,84]. Our results indicate reduced plant taxonomic diversity shortly after grazing exclusion (2–3 years) for both the alpha and gamma components, compared to continuous and deferred grazing (Fig 6). Previous research focusing on the effect of grazing on beta diversity showed both positive [85] and negative [12,86,87] effects, as well as the suggestion that this effect is dependent on the productivity of the system [18]. We found no effect of grazing exclusion on beta taxonomic diversity after the first year of experiment, as well as positive effects of deferred grazing, which is in agreement with results found for Uruguayan productive grasslands [18], and suggests that these systems are able to maintain the spatial heterogeneity of species composition in the absence of management (at least in a four-year time window).

The effect of grazing exclusion on plant functional diversity contrasted to continuous grazing was negative in almost all years of the experiment, for all spatial components (alpha, beta and gamma; Fig 5). Since a functional approach can be useful to address questions related to ecosystem processes [88] and services [7], we calculated functional diversity using plant life form traits, which reflect different strategies of habitat occupancy and response to disturbances. Therefore, the resulting values of functional diversity indicated how well distributed was the relative contribution of the different life forms in a given community (subplot or plot, depending on the spatial component). The negative effect of grazing exclusion on functional diversity that we report here is an indication of structural homogenization (i.e., dominance of specific life forms), which agrees with the results reported for taxonomic diversity. However, we did not observe the same increase in functional diversity after the first year of exclusion, suggesting that the functional structure suffered from homogenization faster, and did not benefit from the reduction of dominance promoted immediately after grazing exclusion. In fact, what we observed regarding functional composition was a very fast shift of dominance from a horizontal to a vertical strategy of occupancy. Surprisingly, the effect of deferred grazing on plant functional diversity was very similar. Although the deferred grazing treatment promoted a positive effect on the diversity of plant species, the same effect was not observed for the functional dimension of the community, indicating that a functionally similar (but taxonomically independent) set of species benefited from the alleviation of grazing pressure. Congruent results have been reported for Uruguayan grasslands, with reduction or cessation of grazing promoting fast floristic shifts that benefited erect and tall grasses [39].

### Arthropods

The effects of both deferred grazing and grazing exclusion treatments cascaded through the animal communities inhabiting the grassland vegetation, promoting an overall increase of arthropod high-taxa richness, and abundance of individuals from different trophic levels, i.e. from herbivores such as true bugs (Hemiptera) and flies (Diptera), to predators such as spiders (Araneae) (Fig 7A). Vegetation is the nutritional base for arthropod food webs, as well as the physical habitat structure where they shelter, forage and reproduce [24]. As such, we could draw several mechanisms, probably acting simultaneously, by which grazing alleviation or exclusion modify plant community structure and directly drive arthropod communities (see [25]). One example is the "more individuals hypothesis", that refers to the increase in resource quantity (e.g., food biomass) mediating the increase in total consumer abundance and diversity [89,90]. One of the clearest grazing exclusion effect we found was the increase in spider abundance in all years of the experiment, corroborating previously expected patterns [91], and indicating a plausible relation of spiders with prey abundance and vegetation architecture (tussock grasses and lignified plants; [92]). These finding suggest possible consequences for ecological functions mediated by arthropods in the ecosystems, such as herbivory and predation rates, which has been shown to be influenced by herbivore and predator abundances in grasslands [93,94]. Further, ungrazed or lightly grazed grasslands may be also resource-rich environments even for higher trophic levels of the ecosystem, such as birds, since the availability of vegetation arthropods is considered a key factor on the mechanisms that link grazing management to changes in bird populations [95].

Conversely, we found no consistent effects of deferred grazing or grazing exclusion on epigeic arthropod communities (Fig 7B). Such organisms are usually very sensitive to grassland management (e.g., [96]), relying on the variation of several soil attributes mediated by defoliation, defecation and trampling (e.g., [24]). Here, our high-taxa resolution approach was not able to detect such management effects, indicating that continuous grazing, deferred grazing and grazing exclusion support similar epigeic arthropod communities in terms of higher-taxa richness and abundance. ‘Top-down’ taxonomic approaches, as we used here, in which biodiversity is compared among groups of sites based on higher taxa (e.g., order, family, genus; [40]), present several advantages related to reduction of survey costs and speed, and is usually useful to reveal general community-wide patterns [26]. However, it certainly has obscured some interesting and taxa-specific results that remained undetectable at this resolution, and will be explored in detail in future studies.

### Conclusions

Our results obtained during the first five years of a long-term experiment indicated that the alleviation of grazing by deferred grazing and grazing exclusion promoted detectable changes in a continuously grazed grassland community. The overall effect of grazing exclusion on plant taxonomic diversity was negative. Conversely, deferred grazing promoted an increase in plant taxonomic diversity. Both treatments promoted negative effects on plant functional diversity. We found that the alleviation of grazing promoted the break of dominance by prostrate species, followed by a surprisingly fast homogenization of vegetation structure towards a state with dominance of ligneous and erect species. These management-induced grassland habitat changes led to increases in high-taxa richness and abundance of vegetation-dwelling arthropod groups under both deferred grazing and grazing exclusion, but had no clear detectable effects on epigeic arthropods. These results combined suggest that conservation of southern Brazil grasslands should maintain patches of both intensive and alleviated levels of grazing management (and other disturbances such as fire), but not complete grazing exclusion, to maximize conservation results when considering plant and arthropod communities. However, since grassland ecosystem conservation in protected areas is nearly insignificant [33,97], and management including disturbances such as fire and cattle grazing in protected areas is still a taboo issue in Brazil [98], grasslands harboring such conditions can be found mostly in private properties. This emphasizes the important role of scientists and policy-makers in encouraging and providing technical tools for farmers to manage natural grasslands in a way to simultaneously conserve biodiversity and achieve more productive forage source to grazing animals, which as pointed out by [99] can avoid land conversion.

In practical terms, our results indicate that either a reduction of stocking rates or the introduction of rotational grazing are beneficial to biodiversity conservation, while they can also increase biomass production and thus productivity [44]. In our experimental design, deferred grazing was applied in small plots that allowed us to study the biological responses of the system. This is still only a first step towards the development of farm-level conservation and management strategies. However, land owners, usually with a production-oriented perspective, showed high interest in the results, and at one study site an experimental study working in larger paddocks has already been implemented.

## Supporting information

S1 Table Description and location of the six sampling sites. All sites comprise natural grassland areas under cattle grazing, in which the experimental blocks were assembled.
(DOCX)

S2 Table List of vascular plants present in six sampling sites across 5 years of sampling (n = 441), with respective botanical families and life form categories. List of vascular plants present in six sampling sites across 5 years of sampling (n = 441), with respective botanical families and life form categories. bg = bulbous geophyte, ct = connected tussock, de = decumbent, hf = herbaceous forb, lf = lignified forb, rh = rhizomatous, ro = rosette, sh = shrub, ss = subshrub, st = stoloniferous, su = succulent, te = solitary tussock, th = therophyte. See Fig 2 in the main text for life form descriptions and a key for classifying species.
(DOCX)

S3 Table Relative cover values of plant life forms across in each site, year and treatment. Sites: ACE = Aceguá municipality, ALE = Alegrete municipality, ARA = Aratinga Ecological Station, LAV = Lavras do Sul municipality, TAI = Tainhas State Park, APA = Aparados da Serra National Park. Treatments refer to continuous or differed (intermittent) grazing, and grazing exclusion. Plant life forms: bg = bulbous geophyte, ct = connected tussock, de = decumbent, hf = herbaceous forb, lf = lignified forb, rh = rhizomatous, ro = rosette, sh = shrub, ss = subshrub, st = stoloniferous, su = succulent, te = solitary tussock, th = therophyte.
(DOCX)

S4 Table Abundance of individuals: Arthropods from grassland vegetation. Sites: ACE = Aceguá municipality, ALE = Alegrete municipality, ARA = Aratinga Ecological Station, LAV = Lavras do Sul municipality, TAI = Tainhas State Park, APA = Aparados da Serra National Park. Treatments refer to continuous or differed (intermittent) grazing, and grazing exclusion.
(DOCX)

S5 Table Abundance of individuals: Epigeic arthropods. Sites: ACE = Aceguá municipality, ALE = Alegrete municipality, ARA = Aratinga Ecological Station, LAV = Lavras do Sul municipality, TAI = Tainhas State Park, APA = Aparados da Serra National Park. Treatments refer to continuous or differed (intermittent) grazing, and grazing exclusion.
(DOCX)

S1 Fig Illustration of the sampling procedures. A. Plant community survey in 1m^2^ subplots (alpha component; n = 162). B. Biomass sampling. C. Pitfall trap used to sample epigeic arthropods. D. Sweeping nets used to sample vegetation arthropods.
(TIF)
