# Correction: Long-term ecological research in southern Brazil grasslands: Effects of grazing exclusion and deferred grazing on plant and arthropod communities

**DOI:** 10.1371/journal.pone.0228886

**Published:** 2020-02-03

**Authors:** The PLOS ONE Staff

There are errors in the author affiliations. The correct affiliations are as follows:

Pedro M.A. Ferreira^1^, Bianca O. Andrade^2,3^, Luciana R. Podgaiski^4^, Amanda C. Dias^4^, Valério D. Pillar^4^, Gerhard E. Overbeck^2,4^, Milton de S. Mendonça Jr.^5^ & Ilsi I. Boldrini^2^

**1** Programa de Pós-Graduação em Ecologia e Evolução da Biodiversidade, Pontifícia Universidade Católica do Rio Grande do Sul, Porto Alegre, RS, Brazil, **2** Departamento de Botânica, Universidade Federal do Rio Grande do Sul, Porto Alegre, RS, Brazil, **3** Department of Agronomy and Horticulture, University of Nebraska, Lincoln, NE, USA, **4** Departamento de Ecologia, Universidade Federal do Rio Grande do Sul, Porto Alegre, RS, Brazil, **5** Programa de Pós-Graduação em Biologia Animal, Universidade Federal do Rio Grande do Sul, Porto Alegre, RS, Brazil.

The publisher apologizes for the errors.
