# Correction: Long-term ecological research in southern Brazil grasslands: Effects of grazing exclusion and deferred grazing on plant and arthropod communities

**DOI:** 10.1371/journal.pone.0229219

**Published:** 2020-02-12

**Authors:** The PLOS ONE Staff

There are errors in the author affiliations. The correct affiliations are as follows:

Pedro M. A. Ferreira^1^, Bianca O. Andrade^2,5^, Luciana R. Podgaiski^3^, Amanda C. Dias^4^, Valério D. Pillar^3^, Gerhard E. Overbeck^2,3^, Milton de S. Mendonça Jr^3^, Ilsi I. Boldrini^2^

**1** Programa de Pós-Graduação em Ecologia e Evolução da Biodiversidade, Pontifícia Universidade Católica do Rio Grande do Sul, Porto Alegre, Rio Grande do Sul, Brazil, **2** Departamento de Botânica, Universidade Federal do Rio Grande do Sul, Porto Alegre, Rio Grande do Sul, Brazil, **3** Departamento de Ecologia, Universidade Federal do Rio Grande do Sul, Porto Alegre, Rio Grande do Sul, Brazil, **4** Programa de Pós-Graduação em Biologia Animal, Universidade Federal do Rio Grande do Sul, Porto Alegre, Rio Grande do Sul, Brazil, **5** Department of Agronomy and Horticulture, University of Nebraska, Lincoln, Nebraska, United States of America.

The publisher apologizes for the errors.
