# Supplementary material for: Long-term ecological research in southern Brazil grasslands: Effects of grazing exclusion and deferred grazing on plant and arthropod communities
Source: PLoS One. 2020 Jan 13;15(1):e0227706. doi: 10.1371/journal.pone.0227706 (PMC6957338; doi:10.1371/journal.pone.0227706)
Supplement: S1 Table — All sites comprise natural grassland areas under cattle grazing, in which the experimental blocks were assembled. (DOCX) [file pone.0227706.s001.docx]

| **Site/ Municipality** | **Coordinates** | **Altitude**  **(m a.s.l.)** | **Temperature (^o^C)** | **Precipitation (mm)** | **Biome** | **Soil classification** |
| --- | --- | --- | --- | --- | --- | --- |
| Aparados da Serra National Park (APA)  São Francisco de Paula | 29^o^08’10”S, 50^o^09’21”W | 924 | 15.2 | 1898 | Highland grasslands | Humic Dystrudept |
| Aratinga Ecological Station (ARA)  Cambará do Sul | 29^o^23’31”S, 50^o^14’30”W | 900 | 15.1 | 2002 | Highland grasslands | Humic Dystrudept |
| Tainhas State Park (TAI)  Jaquirana | 29^o^05’40”S, 50^o^22’03”W | 843 | 15.7 | 1931 | Highland grasslands | Lithic Udorthent |
| Aceguá municipality (ACE)  Aceguá | 31^o^38’55”S, 54^o^09’26”W | 163 | 17.9 | 1152 | Pampa | Vertic Hapludalf |
| Alegrete municipality (ALE)  Alegrete | 30^o^04’08”S, 55^o^59’27”W | 189 | 18.6 | 1507 | Pampa | Typic Udorthent |
| Lavras do Sul municipality (LAV)  Lavras do Sul | 30^o^41’55”S, 53^o^58’11”W | 334 | 17.9 | 1449 | Pampa | Cromic Hapludalf |
