# Supplementary material for: Long-term ecological research in southern Brazil grasslands: Effects of grazing exclusion and deferred grazing on plant and arthropod communities
Source: PLoS One. 2020 Jan 13;15(1):e0227706. doi: 10.1371/journal.pone.0227706 (PMC6957338; doi:10.1371/journal.pone.0227706)
Supplement: S2 Table — List of vascular plants present in six sampling sites across 5 years of sampling (n = 441), with respective botanical families and life form categories. bg = bulbous geophyte, ct = connected tussock, de = decumbent, hf = herbaceous forb, lf = lignified forb, rh = rhizomatous, ro = rosette, sh = shrub, ss = subshrub, st = stoloniferous, su = succulent, te = solitary tussock, th = therophyte. See Fig 2 in the main text for life form descriptions and a key for classifying species. (DOCX) [file pone.0227706.s002.docx]

| **Family** | **Species** | **Life form** |
| --- | --- | --- |
| Acanthaceae | Ruellia morongii Britton | ro |
| Acanthaceae | Stenandrium diphyllum Nees | ro |
| Acanthaceae | Stenandrium dulce (Cav.) Nees | ro |
| Amaranthaceae | Chenopodium haumanii Urb. | ss |
| Amaranthaceae | Pfaffia gnaphaloides (L. f.) Mart. | lf |
| Amaranthaceae | Pfaffia tuberosa (Spreng.) Hicken | lf |
| Amaryllidaceae | Nothoscordum bivalve Britton | bg |
| Amaryllidaceae | Nothoscordum montevidense Beauverd | bg |
| Anacardiaceae | Schinus polygama (Cav.) Cabrera | sh |
| Apiaceae | Eryngium sp. | ro |
| Apiaceae | Ammoselinum rosengurtii Mathias & Constance | th |
| Apiaceae | Centella asiatica Urb. | st |
| Apiaceae | Cyclospermum leptophyllum (Pers.) Sprague ex Britton & P. Wilson | th |
| Apiaceae | Eryngium ebracteatum Lam. | ro |
| Apiaceae | Eryngium echinatum Urb. | ro |
| Apiaceae | Eryngium eriophorum Cham. & Schltdl. | ro |
| Apiaceae | Eryngium horridum Malme | ro |
| Apiaceae | Eryngium nudicaule Lam. | ro |
| Apocynaceae | Oxypetalum microphyllum Hook. & Arn. | de |
| Araliaceae | Hydrocotyle exigua Malme | st |
| Araliaceae | Hydrocotyle pusilla A. Rich. | st |
| Araliaceae | Hydrocotyle ranunculoides L. f. | rh |
| Aristolochiaceae | Aristolochia sessilifolia (Klotzsch) Duch. | de |
| Asteraceae | Achyrocline alata (Kunth) DC. | lf |
| Asteraceae | Achyrocline satureioides (Lam.) DC. | lf |
| Asteraceae | Acmella bellidioides (Smith) R.K.Jansen | de |
| Asteraceae | Acmella leptophylla (DC.) R.K. Jansen | de |
| Asteraceae | Acmella psilocarpa R. K. Jansen | de |
| Asteraceae | Angelphytum sp. | ss |
| Asteraceae | Aspilia pascalioides Griseb. | de |
| Asteraceae | Baccharis articulata (Lam.) Pers. | ss |
| Asteraceae | Baccharis brevifolia DC. | ss |
| Asteraceae | Baccharis coridifolia Spreng. | ss |
| Asteraceae | Baccharis crispa Spreng. | ss |
| Asteraceae | Baccharis dracunculifolia DC. | sh |
| Asteraceae | Baccharis pentodonta Malme | ss |
| Asteraceae | Baccharis riograndensis L. Teodoro & J. Vidal | ss |
| Asteraceae | Baccharis spicata (Lam.) Baill. | ss |
| Asteraceae | Baccharis subtropicalis G. Heiden | ss |
| Asteraceae | Baccharis tridentata Vahl | sh |
| Asteraceae | Barrosoa candolleana (Hook. & Arn.) R.M. King & H. Rob. | ro |
| Asteraceae | Calea cymosa Less. | ro |
| Asteraceae | Calea uniflora Less. | lf |
| Asteraceae | Calyptocarpus biaristatus (DC.) H. Rob. | th |
| Asteraceae | Chaptalia arechavaletae Hieron. | ro |
| Asteraceae | Chaptalia exscapa (Pers.) Baker | ro |
| Asteraceae | Chaptalia integerrima (Vell.) Burkart | ro |
| Asteraceae | Chaptalia mandonii (Schultz-Bip.) Burkart | ro |
| Asteraceae | Chaptalia piloselloides (Vahl) Baker | ro |
| Asteraceae | Chaptalia runcinata Kunth | ro |
| Asteraceae | Chevreulia acuminata Less. | hf |
| Asteraceae | Chevreulia revoluta A.A. Schneid. & Trevis. | hf |
| Asteraceae | Chevreulia sarmentosa (Pers.) Blake | st |
| Asteraceae | Chromolaena ascendens (Sch. Bip. ex Baker) R.M. King & H. Rob. | lf |
| Asteraceae | Chromolaena hirsuta (Hook. & Arn.) R.M.King & H.Rob. | ss |
| Asteraceae | Chromolaena laevigata (Lam.) R.M. King & H. Rob. | ss |
| Asteraceae | Chrysolaena flexuosa (Sims) H.Rob. | lf |
| Asteraceae | Conyza aff. bonariensis | lf |
| Asteraceae | Conyza bonariensis (L.) Cronquist | th |
| Asteraceae | Conyza primulifolia (Lam.) Cuatrec. & Lourteig | ro |
| Asteraceae | Conyza sumatrensis (Retz.) E. Walker | th |
| Asteraceae | Elephantopus mollis Kunth | lf |
| Asteraceae | Facelis retusa (Lam.) Schultz-Bip. | th |
| Asteraceae | Gamochaeta argentina Cabrera | th |
| Asteraceae | Gamochaeta coarctata (Willd.) Kerguélen | ro |
| Asteraceae | Gamochaeta filaginea (DC.) Cabrera | ro |
| Asteraceae | Holocheilus brasiliensis (L.) Cabrera | ro |
| Asteraceae | Holocheilus illustris (Vell.) Cabrera | ro |
| Asteraceae | Holocheilus sp. | ro |
| Asteraceae | Hypochaeris albiflora (Kuntze) Azevêdo-Gonç. & Matzenb. | ro |
| Asteraceae | Hypochaeris catharinensis Cabrera | ro |
| Asteraceae | Hypochaeris chillensis (Kunth) Britton | ro |
| Asteraceae | Hypochaeris lutea (Vell.) Britton | ro |
| Asteraceae | Hypochaeris megapotamica Cabrera | ro |
| Asteraceae | Jaegeria hirta (Lag.) Less. | lf |
| Asteraceae | Calea sp. | hf |
| Asteraceae | Lessingianthus sellowii (Less.) H. Rob. | lf |
| Asteraceae | Lucilia acutifolia (Less.) ex. Baker | hf |
| Asteraceae | Lucilia nitens Less. | hf |
| Asteraceae | Micropsis spathulata (Pers.) Cabrera | th |
| Asteraceae | Neja filiformis (Spreng.) Nees | ss |
| Asteraceae | Noticastrum decumbens (Baker) Cuatrec. | de |
| Asteraceae | Orthopappus angustifolius (Sw.) Gleason | lf |
| Asteraceae | Pamphalea heterophylla Less. | th |
| Asteraceae | Perezia multiflora (Bonpl.) Less. | ro |
| Asteraceae | Podocoma hieraciifolia (Poir.) Cass. | hf |
| Asteraceae | Pterocaulon alopecuroides (Lam.) DC. | lf |
| Asteraceae | Pterocaulon polypterum (DC) Cabr. | lf |
| Asteraceae | Senecio brasiliensis (Spreng) Less. var. brasiliensis | ss |
| Asteraceae | Senecio conyzifolius Baker | ss |
| Asteraceae | Senecio heterotrichius DC. | lf |
| Asteraceae | Senecio madagascariensis Poir. | th |
| Asteraceae | Senecio selloi (Spreng.) DC. | th |
| Asteraceae | Solidago chilensis Meyen | ss |
| Asteraceae | Soliva sessilis Ruiz & Pav. | th |
| Asteraceae | Sonchus oleraceus L. | ro |
| Asteraceae | Stenachaenium campestre Baker | ro |
| Asteraceae | Stevia lundiana DC. | lf |
| Asteraceae | Symphyotrichum graminifolium (Spreng.) G.L. Nesom | ss |
| Asteraceae | Symphyotrichum squamatum (Spreng.) G.L. Nesom | ss |
| Asteraceae | Trichocline catharinensis Cabrera | ro |
| Asteraceae | Vernonanthura nudiflora (Less.) H. Rob. | ss |
| Asteraceae | Vernonia echioides Less. | ss |
| Asteraceae | Viguiera anchusifolia (DC.) Baker | ss |
| Brassicaceae | Cardamine chenopodiifolia Pers. | de |
| Brassicaceae | Lepidium bonariense L. | th |
| Cactaceae | Parodia haselbergii (F. Haage ex Rümpler) F.H. Brandt | su |
| Cactaceae | Parodia ottonis (Lehm.) N.P. Taylor | su |
| Cactaceae | Parodia sp. | su |
| Calyceraceae | Acicarpha procumbens Less. | de |
| Calyceraceae | Acicarpha tribuloides Juss. | th |
| Campanulaceae | Lobelia camporum Pohl | hf |
| Campanulaceae | Lobelia hederacea Cham. | hf |
| Campanulaceae | Triodanis perfoliata (L.) Nieuwl. | hf |
| Campanulaceae | Wahlenbergia linarioides (Lam.) A. DC. | hf |
| Caryophyllaceae | Cerastium commersonianum DC. | hf |
| Caryophyllaceae | Cerastium glomeratum Thuill. | th |
| Caryophyllaceae | Paronychia brasiliana DC. | de |
| Caryophyllaceae | Polycarpon tetraphyllum (L.) L. | de |
| Caryophyllaceae | Sagina humifusa (Cambess.) Fenzl ex Rohrbach in Martius | th |
| Caryophyllaceae | Spergularia grandis (Pers.) Cambess. | hf |
| Caryophyllaceae | Stellaria media (L.) Vill. | th |
| Cistaceae | Helianthemum brasiliense (Lam.) Pers. | lf |
| Commelinaceae | Commelina platyphylla Klotzsch ex Seub. | hf |
| Convolvulaceae | Convolvulus laciniatus Desr. | de |
| Convolvulaceae | Dichondra macrocalyx Meisn. | st |
| Convolvulaceae | Dichondra sericea Swartz | st |
| Convolvulaceae | Evolvulus sericeus Swartz | de |
| Cyperaceae | Abildgaardia ovata (Burm. f.) Kral | te |
| Cyperaceae | Bulbostylis capillaris (L.) C.B. Clarke | te |
| Cyperaceae | Bulbostylis communis M.G. Lopez & D.A. Simpson | th |
| Cyperaceae | Bulbostylis glaziovii (Boeckeler) C.B. Clarke | te |
| Cyperaceae | Bulbostylis juncoides (Vahl) Kük. ex Osten | te |
| Cyperaceae | Bulbostylis sphaerocephala (Boeck.) C.B. Clarke | te |
| Cyperaceae | Bulbostylis subtilis M.G. Lopez | th |
| Cyperaceae | Carex aureolensis Steud. | te |
| Cyperaceae | Carex longii (Kük.) Luceño & Alves | te |
| Cyperaceae | Carex phalaroides Kunth | te |
| Cyperaceae | Carex sororia Kunth | te |
| Cyperaceae | Cyperus aggregatus (Willd) Endl. | te |
| Cyperaceae | Cyperus hermaphroditus (Jacq.) Standl. | te |
| Cyperaceae | Cyperus reflexus Vahl | te |
| Cyperaceae | Eleocharis bonariensis Nees | ct |
| Cyperaceae | Eleocharis dunensis Kük. | ct |
| Cyperaceae | Eleocharis maculosa (Vahl) Roem. & Schult. | ct |
| Cyperaceae | Eleocharis montana (Kunth) Roem. & Schult. | ct |
| Cyperaceae | Eleocharis nudipes (Kunth) Palla | te |
| Cyperaceae | Eleocharis viridans Kük. | ct |
| Cyperaceae | Fimbristylis campestris R. Trevis. & Ronchi, Helen Nuernberg | te |
| Cyperaceae | Fimbristylis dichotoma (L.) Vahl | te |
| Cyperaceae | Kyllinga brevifolia Rottb. | te |
| Cyperaceae | Kyllinga odorata Vahl | te |
| Cyperaceae | Kyllinga vaginata Lam. | te |
| Cyperaceae | LAV Fimbristylis sp. | te |
| Cyperaceae | Lipocarpha humboldtiana Nees | te |
| Cyperaceae | Rhynchospora barrosiana Guagl. | te |
| Cyperaceae | Rhynchospora brownii Roem. & Schult. | te |
| Cyperaceae | Rhynchospora emaciata (Nees) Boeck. | ct |
| Cyperaceae | Rhynchospora flexuosa C.B. Clarke | te |
| Cyperaceae | Rhynchospora holoschoenoides (Rich.) Herter | te |
| Cyperaceae | Rhynchospora junciformis (Kunth) Boeck. | te |
| Cyperaceae | Rhynchospora megapotamica (A. Spreng.) H. Pfeiff. | rh |
| Cyperaceae | Rhynchospora rugosa (Vahl) Gale | ct |
| Cyperaceae | Rhynchospora setigera Griseb. | ct |
| Cyperaceae | Rhynchospora tenuis Link | ct |
| Cyperaceae | Scleria distans Poir. | ct |
| Cyperaceae | Scleria sellowiana Kunth | ct |
| Droseraceae | Drosera brevifolia Pursh | ro |
| Ericaceae | Gaylussacia brasiliensis (Spreng.) Meisn. | sh |
| Ericaceae | Gaylussacia pseudogaultheria Cham. & Schltdl. | ss |
| Eriocaulaceae | Eriocaulaceae sp. | ro |
| Euphorbiaceae | Euphorbia papillosa A. St.-Hil. | lf |
| Euphorbiaceae | Euphorbia peperomioides Boiss. | de |
| Euphorbiaceae | Euphorbia selloi (Klotzsch & Garcke) Boiss. | de |
| Euphorbiaceae | Euphorbia stenophylla (Klotzsch & Garcke) Boiss. | de |
| Euphorbiaceae | Tragia bahiensis Müll. Arg. | de |
| Euphorbiaceae | Tragia uberabana Müll. Arg. | de |
| Fabaceae | Adesmia ciliata Vogel | lf |
| Fabaceae | Adesmia incana Vogel | lf |
| Fabaceae | Aeschynomene falcata (Poir.) DC. | lf |
| Fabaceae | Crotalaria hilariana Benth. | lf |
| Fabaceae | Desmanthus virgatus (L.) Willd. | lf |
| Fabaceae | Desmodium adscendens (Sw.) DC. | lf |
| Fabaceae | Desmodium craspediferum A.M.G. Azevedo & M.L.A.A. Oliveira | lf |
| Fabaceae | Desmodium incanum DC. | lf |
| Fabaceae | Desmodium triarticulatum Malme | lf |
| Fabaceae | Desmodium uncinatum (Jacq.) DC. | lf |
| Fabaceae | Eriosema tacuaremboense Arechav. | lf |
| Fabaceae | Galactia gracillima Benth. | lf |
| Fabaceae | Galactia marginalis Benth. | lf |
| Fabaceae | Galactia neesii DC. | lf |
| Fabaceae | Galactia pretiosa Burkart | lf |
| Fabaceae | Lathyrus subulatus Lam. | lf |
| Fabaceae | Mimosa sp. | lf |
| Fabaceae | Macroptilium prostratum (Benth.) Urb. | lf |
| Fabaceae | Macroptilium psammodes (Lindm.) S.I. Drewes & R.A. Palacios | lf |
| Fabaceae | Rhynchosia corylifolia Mart. ex Benth. | de |
| Fabaceae | Rhynchosia diversifolia Micheli | de |
| Fabaceae | Rhynchosia senna Gillies ex Hook. & Arn. | de |
| Fabaceae | Stylosanthes leiocarpa Vogel | lf |
| Fabaceae | Stylosanthes montevidensis Vogel | lf |
| Fabaceae | Tephrosia adunca Benth. | lf |
| Fabaceae | Trifolium polymorphum Poir. | st |
| Fabaceae | Trifolium riograndense Burkart | st |
| Fabaceae | Vigna peduncularis Fawc. & Rendle | lf |
| Fabaceae | Zornia burkartii Vanni | lf |
| Fabaceae | Zornia orbiculata Mohlenbr. | lf |
| Fabaceae | Zornia ramboiana Mohlenbr. | lf |
| Hypoxidaceae | Hypoxis decumbens L. | bg |
| Iridaceae | Sisyrinchium sp.2 | hf |
| Iridaceae | Calydorea alba Roitman & A. Castillo | bg |
| Iridaceae | Herbertia lahue (Molina) Goldblatt | bg |
| Iridaceae | Herbertia pulchella Sweet | bg |
| Iridaceae | Sisyrinchium megapotamicum Malme | bg |
| Iridaceae | Sisyrinchium micranthum Cav. | th |
| Iridaceae | Sisyrinchium palmifolium L. | hf |
| Iridaceae | Sisyrinchium platense I.M. Johnst. | hf |
| Iridaceae | Sisyrinchium sellowianum Klatt | hf |
| Iridaceae | Sisyrinchium vaginatum Spreng. | hf |
| Iridaceae | Sisyrinchium sp. | hf |
| Juncaceae | Juncus bufonius L. | th |
| Juncaceae | Juncus capillaceus Lam. | te |
| Juncaceae | Juncus microcephalus Kunth | te |
| Juncaceae | Juncus tenuis Willd. | te |
| Juncaceae | Luzula ulei Buchenau | ct |
| Lamiaceae | Condea elegans (Briq.) Harley & J.F.B. Pastore | ss |
| Lamiaceae | Cunila galioides Benth. | lf |
| Lamiaceae | Glechon ciliata Benth. | lf |
| Lamiaceae | Rhabdocaulon gracile (Benth.) Epling | lf |
| Lamiaceae | Salvia ovalifolia A. St.-Hil. | ss |
| Lamiaceae | Scutellaria racemosa Pers. | hf |
| Linaceae | Cliococca selaginoides (Lam.) C.M. Rogers & Mildnerso | hf |
| Lythraceae | Cuphea glutinosa Cham. & Schltdl. | lf |
| Lythraceae | Heimia apetala (Spreng.) S.A. Graham & Gandhi | sh |
| Malvaceae | Ayenia mansfeldiana (Herter) Herter & Cristobal | lf |
| Malvaceae | Krapovickasia flavescens (Cav.) Fryxell | lf |
| Malvaceae | Krapovickasia macrodon (DC.) Fryxell | lf |
| Malvaceae | Modiolastrum sp. | st |
| Malvaceae | Pavonia glechomoides A. St.-Hil. | ss |
| Malvaceae | Sida rhombifolia L. | ss |
| Malvaceae | Melochia sp. | lf |
| Malvaceae | Turnera sidoides L. | lf |
| Melastomataceae | Rhynchanthera brachyrhyncha Cham. | ss |
| Melastomataceae | Tibouchina gracilis Cogn. | ss |
| Moraceae | Dorstenia brasiliensis Lam. | ro |
| Myrtaceae | Campomanesia aurea O. Berg | sh |
| Onagraceae | Oenothera parodiana Munz | th |
| Ophioglossaceae | Ophioglossum sp. | hf |
| Ophioglossaceae | Ophioglossum crotalophoroides | hf |
| Orchidaceae | Habenaria parviflora Lindl. | hf |
| Orchidaceae | Skepterostachys sp. | ro |
| Orchidaceae | Skeptrostachys arechavaletanii (Barb. Rodr.) Garay | ro |
| Orchidaceae | Skeptrostachys balanophorostachya (Rchb. f. ex Warm.) Garay | ro |
| Orobanchaceae | Agalinis communis (Cham. & Schltdl.) D'Arcy | th |
| Orobanchaceae | Buchnera longifolia Kunth | lf |
| Oxalidaceae | Oxalis articulata Savigny | bg |
| Oxalidaceae | Oxalis bipartita A. St.-Hil. | bg |
| Oxalidaceae | Oxalis brasiliensis Lodd. | bg |
| Oxalidaceae | Oxalis conorrhiza Jacq. | bg |
| Oxalidaceae | Oxalis debilis Kunth | bg |
| Oxalidaceae | Oxalis eriocarpa DC. | bg |
| Oxalidaceae | Oxalis lasiopetala Zucc. | bg |
| Oxalidaceae | Oxalis perdicaria (Molina) Bertero | bg |
| Plantaginaceae | Berroa gnaphalioides (Less.) Beauverd | de |
| Plantaginaceae | Gratiola peruviana L. | hf |
| Plantaginaceae | Mecardonia procumbens (Mill.) Small | de |
| Plantaginaceae | Plantago australis Lam. | ro |
| Plantaginaceae | Plantago myosuros Lam. | th |
| Plantaginaceae | Plantago penantha Griseb. | th |
| Plantaginaceae | Plantago tomentosa Lam. | ro |
| Plantaginaceae | Scoparia dulcis L. | ss |
| Plantaginaceae | Stemodia verticillata (Mill.) Hassl. | hf |
| Plantaginaceae | Veronica arvensis L. | th |
| Poaceae | Agrostis hygrometrica Nees | te |
| Poaceae | Agrostis lenis Roseng., B.R. Arrill. & Izag. | te |
| Poaceae | Agrostis montevidensis Spreng. ex Nees | te |
| Poaceae | Agrostis tandilensis (Kuntze) Parodi | th |
| Poaceae | Andropogon lateralis Nees | te |
| Poaceae | Andropogon macrothrix Trin. | te |
| Poaceae | Andropogon ternatus (Spreng.) Nees | te |
| Poaceae | Anthaenantia lanata (Kunth) Benth. | te |
| Poaceae | Aristida echinulata Roseng. & Izag. | te |
| Poaceae | Aristida flaccida Trin. & Rupr. | te |
| Poaceae | Aristida laevis (Nees) Kunth | te |
| Poaceae | Aristida murina Cav. | te |
| Poaceae | Aristida spegazzinii Arechav. | te |
| Poaceae | Aristida uruguayensis Henrard | te |
| Poaceae | Aristida venustula Arechav. | te |
| Poaceae | Axonopus affinis Chase | st |
| Poaceae | Axonopus argentinus Parodi | te |
| Poaceae | Axonopus compressus (Sw.) P. Beauv. | st |
| Poaceae | Axonopus fissifolius (Raddi) Kuhlm. | st |
| Poaceae | Axonopus ramboi G.A. Black | te |
| Poaceae | Axonopus siccus (Nees) Kuhlm. | te |
| Poaceae | Axonopus suffultus (Mikan ex Trin.) Parodi | te |
| Poaceae | Bothriochloa laguroides (DC.) Herter | te |
| Poaceae | Bouteloua megapotamica (Spreng.) Kuntze | ct |
| Poaceae | Briza minor L. | th |
| Poaceae | Bromus auleticus Trin. ex Nees | te |
| Poaceae | Calamagrostis viridiflavescens (Poir.) Steud. | te |
| Poaceae | Chascolytrum brizoides (Lam.) L. Essi, Longhi-Wagner & Souza-Chies | te |
| Poaceae | Chascolytrum calotheca (Trin.) Essi, Longhi-Wagner & Souza-Chies | te |
| Poaceae | Chascolytrum lamarckianum (Nees) Matthei | te |
| Poaceae | Chascolytrum poomorphum (J. Presl.) Essi, Longhi- Wagner & Souza-Chies | te |
| Poaceae | Chascolytrum rufum J. Presl | te |
| Poaceae | Chascolytrum scabrum (Nees ex Steud.) Matthei | te |
| Poaceae | Chascolytrum subaristatum (Lam.) Desv. | te |
| Poaceae | Chascolytrum uniolae (Nees) Essi, Longhi-Wagner & Souza-Chies | te |
| Poaceae | Chloris grandiflora Roseng. & Izag. | te |
| Poaceae | Cynodon dactylon (L.) Pers. | rh |
| Poaceae | Danthonia cirrata Hack. & Arechav. | te |
| Poaceae | Danthonia montana Döll | te |
| Poaceae | Danthonia montevidensis Hack. & Arechav. | te |
| Poaceae | Danthonia secundiflora J. Presl | te |
| Poaceae | Deschampsia cespitosa (L.) P. Beauv. | te |
| Poaceae | Dichanthelium sabulorum (Lam.) Gould & C.A. | de |
| Poaceae | Eleusine tristachya (Lam.) Lam. | te |
| Poaceae | Eragrostis airoides Nees | te |
| Poaceae | Eragrostis bahiensis Schrad. ex Schult. | te |
| Poaceae | Eragrostis lugens Nees | te |
| Poaceae | Eragrostis neesii Trin. | te |
| Poaceae | Eragrostis plana Nees | te |
| Poaceae | Eragrostis polytricha Nees | te |
| Poaceae | Eragrostis retinens Hack. & Arechav. | te |
| Poaceae | Eriochrysis villosa Swallen | te |
| Poaceae | Eustachys brevipila (Roseng. & Izag.) Caro & E.A. Sánchez | te |
| Poaceae | Eustachys uliginosa (Hack.) Herter | te |
| Poaceae | Gymnopogon burchellii (Munro ex Döll) Ekman | te |
| Poaceae | Gymnopogon grandiflorus Roseng., B.R. Arrill. & Izag. | te |
| Poaceae | Hordeum euclaston Steud. | th |
| Poaceae | Lolium multiflorum Lam. | th |
| Poaceae | Melica rigida Cav. | te |
| Poaceae | Microchloa indica (L. f.) P. Beauv. | te |
| Poaceae | Mnesithea selloana (Hack.) de Koning & Sosef | te |
| Poaceae | Nassella filiculmis (Delile) Barkworth | te |
| Poaceae | Nassella juergensii (Hack.) Barkworth | te |
| Poaceae | Nassella melanosperma (J. Presl) Barkworth | te |
| Poaceae | Nassella mucronata (Kunth) R.W. Pohl | te |
| Poaceae | Nassella nutans (Hack.) Barkworth | te |
| Poaceae | Nassella tenuiculmis (Hack.) Peñail. | te |
| Poaceae | Nassella vallsii (A. Zanin & Longhi-Wagner) Peñail. | te |
| Poaceae | Panicum bergii Arechav. | te |
| Poaceae | Paspalum almum Chase | te |
| Poaceae | Paspalum barretoi Canto-Dorow, Valls & Longhi-Wagner | rh |
| Poaceae | Paspalum compressifolium Swallen | te |
| Poaceae | Paspalum dilatatum Poir. | te |
| Poaceae | Paspalum guenoarum Arechav. | te |
| Poaceae | Paspalum indecorum Mez | rh |
| Poaceae | Paspalum lepton Schult. | rh |
| Poaceae | Paspalum modestum Mez | st |
| Poaceae | Paspalum notatum Flüggé | rh |
| Poaceae | Paspalum plicatulum Michx. | te |
| Poaceae | Paspalum polyphyllum Nees ex Trin. | te |
| Poaceae | Paspalum pumilum Nees | rh |
| Poaceae | Paspalum umbrosum Trin. | rh |
| Poaceae | Paspalum urvillei Steud. | te |
| Poaceae | Phalaris platensis Henrard ex Wacht. | te |
| Poaceae | Piptochaetium alpinum L. B. Sm. | te |
| Poaceae | Piptochaetium bicolor (Vahl) E. Desv. | te |
| Poaceae | Piptochaetium lasianthum Griseb. | te |
| Poaceae | Piptochaetium montevidense (Spreng.) Parodi | te |
| Poaceae | Piptochaetium ruprechtianum E. Desv. | te |
| Poaceae | Piptochaetium stipoides (Trin. & Rupr.) Hack. ex Arechav. | te |
| Poaceae | Piptochaetium uruguense Griseb. | te |
| Poaceae | Poa annua L. | th |
| Poaceae | Poa lanigera Swallen | te |
| Poaceae | Saccharum angustifolium (Nees) Trin. | te |
| Poaceae | Sacciolepis vilvoides (Trin.) Chase | te |
| Poaceae | Schizachyrium condensatum (Kunth) Nees | te |
| Poaceae | Schizachyrium gracilipes (Hack.) A. Camus | te |
| Poaceae | Schizachyrium hatschbachii Peichoto | te |
| Poaceae | Schizachyrium imberbe (Hack.) A. Camus | te |
| Poaceae | Schizachyrium microstachyum (Desv. ex Ham.) Roseng., B.R. Arrill. & Izag. | te |
| Poaceae | Schizachyrium spicatum (Spreng.) Herter | te |
| Poaceae | Schizachyrium tenerum Nees | te |
| Poaceae | Setaria fiebrigii R.A.W. Herrm. | te |
| Poaceae | Setaria parviflora (Poir.) Kerguélen | te |
| Poaceae | Setaria vaginata Spreng. | te |
| Poaceae | Sorghastrum pellitum (Hack.) Parodi | te |
| Poaceae | Sorghastrum setosum (Griseb.) Hitchc. | te |
| Poaceae | Sorghastrum stipoides (Kunth) Nash | te |
| Poaceae | Sporobolus camporum Swallen | te |
| Poaceae | Sporobolus indicus (L.) R. Br. | te |
| Poaceae | Sporobolus monandrus Roseng., B.R. Arrill. & Izag. | te |
| Poaceae | Steinchisma decipiens (Nees ex Trin.) W.V. Br. | te |
| Poaceae | Steinchisma hians (Elliott) Nash | te |
| Poaceae | Trachypogon montufarii (Kunth) Nees | te |
| Poaceae | Tridens hackelii (Arechav.) Parodi | te |
| Poaceae | Vulpia bromoides (L.) Gray | th |
| Polygalaceae | Polygala adenophylla A . St-Hil. & Moq. | lf |
| Polygalaceae | Polygala australis A . W. Benn. | th |
| Polygalaceae | Polygala brasiliensis L. | lf |
| Polygalaceae | Polygala linoides Poir. | th |
| Polygalaceae | Polygala molluginifolia A. St.-Hil & Moq. | lf |
| Polygalaceae | Polygala pulchella A. St.-Hil & Moq. | th |
| Primulaceae | Lysimachia minima (L.) U. Manns & Anderb. | th |
| Primulaceae | Lysimachia sp. | th |
| Primulaceae | Pelletiera serpyllifolia (Scherb.) Webb & Berthel | th |
| Ranunculaceae | Anemone decapetala Ard. | hf |
| Ranunculaceae | Ranunculus bonariensis Poir. | hf |
| Rosaceae | Acaena eupatoria Cham. & Schltdl. | de |
| Rosaceae | Aphanes arvensis L. | th |
| Rosaceae | Prunus myrtifolia (L.) Urb. | sh |
| Rubiaceae | Borreria brachystemonoides Cham. & Schltdl. | lf |
| Rubiaceae | Borreria dasycephala (Cham. & Schltdl.) Bacigalupo & E.L. Cabral | lf |
| Rubiaceae | Borreria eryngioides Cham. & Schltdl. | lf |
| Rubiaceae | Borreria tenella (Kunth) Cham. & Schltdl. | lf |
| Rubiaceae | Borreria verticillata (L.) G. Mey. | lf |
| Rubiaceae | Diodella radula (Willd.) Delprete | lf |
| Rubiaceae | Galianthe fastigiata Griseb. | lf |
| Rubiaceae | Galium hirtum Lam. | de |
| Rubiaceae | Galium richardianum (Gillies ex Hook. & Arn.) Endl. ex Walp. | de |
| Rubiaceae | Galium uruguayense Bacigalupo | de |
| Rubiaceae | Galium vile (Cham. & Schltdl.) Dempster | de |
| Rubiaceae | Oldenlandia salzmannii (DC.) Benth. & Hook. f. ex B.D.Jacks | de |
| Rubiaceae | Richardia brasiliensis Gomes | de |
| Rubiaceae | Richardia grandiflora (Cham. & Schltdl.) Steud. | de |
| Rubiaceae | Richardia humistrata (Cham. & Schltdl.) Steud. | de |
| Rubiaceae | Richardia stellaris (Cham. & Schltdl.) Steud. | de |
| Selaginellaceae | Selaginella sp. | de |
| Solanaceae | Bouchetia anomala (Miers) Britton & Rusby | lf |
| Solanaceae | Nierembergia riograndensis Hunz. & A.A. Cocucci | lf |
| Solanaceae | Petunia integrifolia Schinz & Thell. | de |
| Solanaceae | Solanum aculeatissimum Jacq. | ss |
| Solanaceae | Solanum americanum Mill. | ss |
| Solanaceae | Solanum atropurpureum Schrank | ss |
| Solanaceae | Solanum commersonii Dunal | ss |
| Solanaceae | Solanum hasslerianum Chodat | ss |
| Solanaceae | Solanum sp. | ss |
| Verbenaceae | Glandularia aristigera (S. Moore) Tronc. | st |
| Verbenaceae | Glandularia catharinae (Moldenke) N. O'Leary & P. Peralta | st |
| Verbenaceae | Glandularia marrubioides (Cham.) Tronc. | de |
| Verbenaceae | Glandularia selloi (Spreng.) Tronc. | st |
| Verbenaceae | Lippia turnerifolia Cham. | lf |
| Verbenaceae | Lippia villafloridana Kuntze | lf |
| Verbenaceae | Phyla nodiflora (L.) Greene | st |
| Verbenaceae | Verbena gracilescens (Cham.) Herter | lf |
| Verbenaceae | Verbena hirta Spreng. | lf |
| Verbenaceae | Verbena montevidensis Spreng. | lf |
| Verbenaceae | Verbena rigida Spreng. | lf |
| Violaceae | Hybanthus parviflorus (L. f.) Baill. | lf |
