# Supplementary material for: Long-term ecological research in southern Brazil grasslands: Effects of grazing exclusion and deferred grazing on plant and arthropod communities
Source: PLoS One. 2020 Jan 13;15(1):e0227706. doi: 10.1371/journal.pone.0227706 (PMC6957338; doi:10.1371/journal.pone.0227706)
Supplement: S3 Table — Sites: ACE = Aceguá municipality, ALE = Alegrete municipality, ARA = Aratinga Ecological Station, LAV = Lavras do Sul municipality, TAI = Tainhas State Park, APA = Aparados da Serra National Park. Treatments refer to continuous or differed (intermittent) grazing, and grazing exclusion. Plant life forms: bg = bulbous geophyte, ct = connected tussock, de = decumbent, hf = herbaceous forb, lf = lignified forb, rh = rhizomatous, ro = rosette, sh = shrub, ss = subshrub, st = stoloniferous, su = succulent, te = solitary tussock, th = therophyte. (DOCX) [file pone.0227706.s003.docx]

|  |  |  | **Life forms** | | | | | | | | | | | | |
| --- | --- | --- | --- | --- | --- | --- | --- | --- | --- | --- | --- | --- | --- | --- | --- |
| **Site** | **Year** | **Treatment** | **bg** | **ct** | **de** | **hf** | **lf** | **rh** | **ro** | **sh** | **ss** | **st** | **su** | **te** | **th** |
| ACE | 2010 | Continuous | 0.18 | 0.00 | 0.28 | 0.10 | 0.12 | 1.91 | 0.31 | 0.00 | 0.51 | 0.12 | 0.00 | 0.60 | 0.10 |
| ACE | 2010 | Differed | 0.10 | 0.00 | 0.19 | 0.10 | 0.10 | 1.67 | 0.13 | 0.00 | 0.33 | 0.33 | 0.00 | 0.66 | 0.16 |
| ACE | 2010 | Exclusion | 0.16 | 0.10 | 0.23 | 0.10 | 0.20 | 1.89 | 0.15 | 0.10 | 0.10 | 0.28 | 0.00 | 0.60 | 0.13 |
| ACE | 2011 | Continuous | 0.34 | 0.00 | 0.18 | 0.10 | 0.15 | 1.78 | 0.19 | 0.00 | 0.83 | 0.31 | 0.00 | 0.50 | 0.12 |
| ACE | 2011 | Differed | 0.10 | 0.00 | 0.19 | 0.10 | 0.12 | 1.03 | 0.17 | 0.00 | 0.10 | 0.16 | 0.00 | 0.62 | 0.31 |
| ACE | 2011 | Exclusion | 0.10 | 0.00 | 0.12 | 0.30 | 0.21 | 1.15 | 0.13 | 0.00 | 0.18 | 0.21 | 0.00 | 0.63 | 0.29 |
| ACE | 2012 | Continuous | 0.14 | 0.00 | 0.17 | 0.10 | 0.10 | 1.74 | 0.21 | 0.00 | 1.55 | 0.61 | 0.00 | 0.28 | 0.17 |
| ACE | 2012 | Differed | 0.12 | 0.10 | 0.16 | 0.00 | 0.28 | 1.03 | 0.11 | 0.00 | 0.26 | 0.13 | 0.00 | 0.55 | 0.36 |
| ACE | 2012 | Exclusion | 0.22 | 0.00 | 0.18 | 0.00 | 2.03 | 1.18 | 0.18 | 0.00 | 0.93 | 0.38 | 0.00 | 0.81 | 0.10 |
| ACE | 2013 | Continuous | 0.16 | 0.10 | 0.16 | 0.28 | 0.10 | 1.22 | 0.32 | 0.00 | 1.40 | 0.52 | 0.00 | 0.26 | 0.21 |
| ACE | 2013 | Differed | 0.16 | 0.00 | 0.12 | 0.18 | 0.14 | 0.84 | 0.13 | 0.00 | 0.21 | 0.34 | 0.00 | 0.47 | 0.29 |
| ACE | 2013 | Exclusion | 0.38 | 0.50 | 0.10 | 0.10 | 0.23 | 0.64 | 0.10 | 0.00 | 1.55 | 0.17 | 0.00 | 0.95 | 0.10 |
| ACE | 2014 | Continuous | 0.23 | 0.00 | 0.10 | 0.16 | 0.10 | 1.19 | 0.24 | 0.00 | 0.57 | 0.65 | 0.00 | 0.42 | 0.14 |
| ACE | 2014 | Differed | 0.20 | 0.10 | 0.10 | 0.10 | 0.41 | 1.06 | 0.10 | 0.00 | 0.35 | 0.87 | 0.00 | 0.40 | 0.11 |
| ACE | 2014 | Exclusion | 0.10 | 0.00 | 0.10 | 0.00 | 1.23 | 0.16 | 0.33 | 0.10 | 2.06 | 0.50 | 0.00 | 0.50 | 0.10 |
| ALE | 2010 | Continuous | 0.00 | 0.50 | 0.10 | 0.10 | 0.14 | 1.40 | 0.10 | 0.80 | 0.00 | 0.43 | 0.00 | 0.54 | 0.11 |
| ALE | 2010 | Differed | 0.00 | 0.00 | 0.10 | 0.10 | 0.15 | 0.84 | 0.10 | 0.65 | 0.00 | 0.21 | 0.00 | 0.52 | 0.13 |
| ALE | 2010 | Exclusion | 0.00 | 0.00 | 0.18 | 0.10 | 0.24 | 1.11 | 0.10 | 0.67 | 0.00 | 0.24 | 0.00 | 0.50 | 0.10 |
| ALE | 2011 | Continuous | 0.10 | 0.26 | 0.11 | 0.10 | 0.32 | 2.84 | 0.12 | 0.58 | 0.00 | 0.28 | 0.00 | 0.31 | 0.10 |
| ALE | 2011 | Differed | 0.10 | 0.00 | 0.10 | 0.10 | 0.29 | 1.29 | 0.10 | 0.48 | 0.00 | 0.19 | 0.00 | 0.54 | 0.11 |
| ALE | 2011 | Exclusion | 0.10 | 0.10 | 0.13 | 0.10 | 0.47 | 1.67 | 0.10 | 0.57 | 0.00 | 0.14 | 0.00 | 0.52 | 0.17 |
| ALE | 2012 | Continuous | 0.10 | 0.48 | 0.21 | 0.16 | 0.14 | 2.61 | 0.16 | 0.76 | 0.00 | 0.36 | 0.00 | 0.28 | 0.17 |
| ALE | 2012 | Differed | 0.13 | 0.10 | 0.16 | 0.19 | 0.18 | 0.38 | 0.15 | 0.76 | 0.00 | 0.20 | 0.00 | 0.49 | 0.35 |
| ALE | 2012 | Exclusion | 0.10 | 0.00 | 0.14 | 0.22 | 0.27 | 0.61 | 0.10 | 0.98 | 0.00 | 0.25 | 0.00 | 0.71 | 0.52 |
| ALE | 2013 | Continuous | 0.27 | 0.30 | 0.19 | 0.15 | 0.26 | 2.32 | 0.11 | 1.05 | 0.00 | 0.55 | 0.00 | 0.39 | 0.16 |
| ALE | 2013 | Differed | 0.10 | 0.00 | 0.10 | 0.10 | 0.35 | 0.67 | 0.10 | 0.81 | 0.00 | 0.37 | 0.00 | 0.65 | 0.19 |
| ALE | 2013 | Exclusion | 0.10 | 0.00 | 0.15 | 0.10 | 0.27 | 0.31 | 0.10 | 1.64 | 0.00 | 0.16 | 0.00 | 0.76 | 0.10 |
| ALE | 2014 | Continuous | 0.10 | 0.37 | 0.12 | 0.10 | 0.16 | 1.88 | 0.14 | 0.36 | 0.00 | 0.75 | 0.00 | 0.28 | 0.17 |
| ALE | 2014 | Differed | 0.10 | 0.10 | 0.12 | 0.10 | 0.15 | 1.01 | 0.10 | 0.61 | 0.00 | 0.54 | 0.00 | 0.45 | 0.13 |
| ALE | 2014 | Exclusion | 0.10 | 0.00 | 0.16 | 0.10 | 0.10 | 0.37 | 0.10 | 0.60 | 0.00 | 0.10 | 0.00 | 0.84 | 0.29 |
| ARA | 2010 | Continuous | 0.10 | 0.23 | 0.10 | 0.10 | 0.10 | 0.53 | 0.32 | 0.10 | 0.00 | 0.61 | 0.00 | 1.05 | 0.20 |
| ARA | 2010 | Differed | 0.10 | 0.35 | 0.10 | 0.31 | 0.10 | 0.30 | 0.22 | 0.21 | 0.00 | 0.65 | 0.00 | 0.88 | 0.18 |
| ARA | 2010 | Exclusion | 0.10 | 0.54 | 0.10 | 0.20 | 0.10 | 0.20 | 0.43 | 0.24 | 0.00 | 0.35 | 0.00 | 0.85 | 0.10 |
| ARA | 2011 | Continuous | 0.10 | 0.38 | 0.13 | 0.10 | 0.10 | 0.40 | 0.39 | 0.10 | 0.00 | 0.46 | 0.00 | 1.27 | 0.10 |
| ARA | 2011 | Differed | 0.10 | 1.00 | 0.10 | 0.14 | 0.13 | 0.28 | 0.16 | 0.12 | 0.00 | 0.62 | 0.00 | 0.89 | 0.10 |
| ARA | 2011 | Exclusion | 0.10 | 1.12 | 0.10 | 0.10 | 0.10 | 0.10 | 0.32 | 0.28 | 0.00 | 0.31 | 0.00 | 0.98 | 0.10 |
| ARA | 2012 | Continuous | 0.10 | 0.15 | 0.14 | 0.10 | 0.10 | 0.10 | 0.40 | 0.10 | 0.00 | 0.32 | 0.00 | 1.21 | 0.18 |
| ARA | 2012 | Differed | 0.10 | 0.21 | 0.10 | 0.10 | 0.13 | 0.26 | 0.17 | 0.14 | 0.00 | 0.48 | 0.00 | 0.94 | 0.10 |
| ARA | 2012 | Exclusion | 0.10 | 0.47 | 0.10 | 0.10 | 0.13 | 0.10 | 0.23 | 0.32 | 0.00 | 0.20 | 0.00 | 1.05 | 0.00 |
| ARA | 2013 | Continuous | 0.10 | 0.26 | 0.17 | 0.13 | 0.20 | 0.20 | 0.30 | 0.10 | 0.00 | 0.52 | 0.00 | 1.21 | 0.20 |
| ARA | 2013 | Differed | 0.10 | 0.30 | 0.10 | 0.10 | 0.17 | 0.30 | 0.49 | 0.15 | 0.00 | 0.48 | 0.00 | 0.99 | 0.10 |
| ARA | 2013 | Exclusion | 0.10 | 0.52 | 0.10 | 0.10 | 0.10 | 0.00 | 0.25 | 0.38 | 0.00 | 0.10 | 0.00 | 1.60 | 0.10 |
| ARA | 2014 | Continuous | 0.10 | 0.30 | 0.13 | 0.10 | 0.10 | 0.10 | 0.15 | 0.13 | 0.00 | 0.44 | 0.00 | 1.02 | 0.10 |
| ARA | 2014 | Differed | 0.10 | 0.36 | 0.10 | 0.10 | 0.17 | 0.10 | 0.23 | 0.15 | 0.00 | 0.41 | 0.00 | 0.93 | 1.08 |
| ARA | 2014 | Exclusion | 0.10 | 0.29 | 0.10 | 0.10 | 0.10 | 0.10 | 0.72 | 0.30 | 0.00 | 0.50 | 0.00 | 0.99 | 0.10 |
| LAV | 2010 | Continuous | 0.16 | 0.00 | 0.36 | 0.10 | 0.15 | 3.02 | 0.10 | 0.00 | 1.25 | 0.31 | 0.00 | 0.24 | 0.16 |
| LAV | 2010 | Differed | 0.16 | 0.00 | 0.15 | 0.23 | 0.20 | 2.79 | 0.15 | 0.00 | 0.58 | 0.64 | 0.00 | 0.30 | 0.10 |
| LAV | 2010 | Exclusion | 0.10 | 0.00 | 0.10 | 0.10 | 0.21 | 3.11 | 0.10 | 0.00 | 0.91 | 0.45 | 0.00 | 0.28 | 0.13 |
| LAV | 2011 | Continuous | 0.10 | 0.00 | 0.39 | 0.13 | 0.16 | 2.23 | 0.16 | 0.00 | 1.20 | 0.40 | 0.00 | 0.19 | 0.10 |
| LAV | 2011 | Differed | 0.10 | 0.00 | 0.51 | 0.23 | 0.22 | 0.45 | 0.66 | 0.00 | 1.44 | 0.26 | 0.00 | 0.49 | 0.19 |
| LAV | 2011 | Exclusion | 0.16 | 0.00 | 0.36 | 0.10 | 0.18 | 0.63 | 0.10 | 0.00 | 1.72 | 0.24 | 0.00 | 0.45 | 0.18 |
| LAV | 2012 | Continuous | 0.34 | 0.00 | 0.38 | 0.10 | 0.12 | 2.52 | 0.19 | 0.00 | 1.07 | 0.52 | 0.00 | 0.21 | 0.42 |
| LAV | 2012 | Differed | 0.21 | 0.00 | 0.36 | 0.17 | 0.14 | 0.35 | 0.32 | 0.00 | 1.23 | 0.18 | 0.00 | 0.51 | 0.33 |
| LAV | 2012 | Exclusion | 0.18 | 0.00 | 0.51 | 0.10 | 0.14 | 0.42 | 0.14 | 0.00 | 1.58 | 0.33 | 0.00 | 0.61 | 0.20 |
| LAV | 2013 | Continuous | 0.34 | 0.00 | 0.28 | 0.16 | 0.17 | 2.18 | 0.20 | 0.00 | 1.05 | 0.76 | 0.00 | 0.22 | 0.21 |
| LAV | 2013 | Differed | 0.39 | 0.00 | 0.26 | 0.10 | 0.10 | 0.57 | 0.34 | 0.10 | 1.09 | 0.39 | 0.00 | 0.62 | 0.18 |
| LAV | 2013 | Exclusion | 0.19 | 0.00 | 0.23 | 0.10 | 0.15 | 0.10 | 0.21 | 0.10 | 1.63 | 0.21 | 0.00 | 0.51 | 0.18 |
| LAV | 2014 | Continuous | 0.12 | 0.10 | 0.25 | 0.10 | 0.21 | 2.56 | 0.15 | 0.00 | 0.14 | 0.67 | 0.00 | 0.20 | 0.10 |
| LAV | 2014 | Differed | 0.24 | 1.00 | 0.14 | 0.10 | 0.14 | 0.54 | 0.17 | 0.00 | 1.09 | 0.79 | 0.00 | 0.57 | 0.16 |
| LAV | 2014 | Exclusion | 0.10 | 0.00 | 0.10 | 0.10 | 0.12 | 0.00 | 0.10 | 0.00 | 2.31 | 0.30 | 0.00 | 0.48 | 0.10 |
| TAI | 2010 | Continuous | 0.10 | 0.50 | 0.13 | 0.14 | 0.18 | 1.16 | 0.10 | 0.10 | 0.40 | 0.21 | 0.00 | 0.57 | 0.10 |
| TAI | 2010 | Differed | 0.00 | 1.37 | 0.14 | 0.10 | 0.20 | 0.85 | 0.17 | 0.00 | 0.19 | 0.14 | 0.10 | 0.69 | 0.10 |
| TAI | 2010 | Exclusion | 0.10 | 0.23 | 0.15 | 0.10 | 0.17 | 0.16 | 0.10 | 0.00 | 0.30 | 0.27 | 0.00 | 0.78 | 0.10 |
| TAI | 2011 | Continuous | 0.00 | 1.03 | 0.31 | 0.16 | 0.18 | 0.35 | 0.10 | 0.00 | 0.51 | 0.24 | 0.10 | 0.76 | 0.10 |
| TAI | 2011 | Differed | 0.10 | 0.53 | 0.16 | 0.10 | 0.12 | 0.38 | 0.16 | 0.00 | 0.47 | 0.34 | 0.00 | 0.86 | 0.10 |
| TAI | 2011 | Exclusion | 0.00 | 0.00 | 0.17 | 0.10 | 0.16 | 0.23 | 0.10 | 0.00 | 0.40 | 0.24 | 0.00 | 0.80 | 0.10 |
| TAI | 2012 | Continuous | 0.00 | 0.55 | 0.21 | 0.10 | 0.16 | 0.32 | 0.16 | 0.00 | 0.33 | 0.32 | 0.10 | 0.92 | 0.23 |
| TAI | 2012 | Differed | 0.10 | 1.07 | 0.14 | 0.10 | 0.16 | 0.47 | 0.20 | 0.00 | 0.41 | 0.14 | 0.00 | 1.00 | 0.10 |
| TAI | 2012 | Exclusion | 0.10 | 0.00 | 0.32 | 0.10 | 0.17 | 0.26 | 0.50 | 0.00 | 0.34 | 0.29 | 0.00 | 0.88 | 0.10 |
| TAI | 2013 | Continuous | 0.10 | 0.10 | 0.18 | 0.10 | 0.18 | 0.36 | 0.10 | 0.00 | 0.23 | 0.28 | 0.10 | 0.89 | 0.10 |
| TAI | 2013 | Differed | 0.10 | 0.87 | 0.20 | 0.10 | 0.15 | 0.33 | 0.23 | 0.10 | 0.59 | 0.22 | 0.00 | 0.85 | 0.10 |
| TAI | 2013 | Exclusion | 0.10 | 0.10 | 0.17 | 0.10 | 0.13 | 0.10 | 0.10 | 0.00 | 0.33 | 0.10 | 0.00 | 0.83 | 0.10 |
| TAI | 2014 | Continuous | 0.10 | 0.10 | 0.14 | 0.10 | 0.17 | 0.69 | 0.30 | 0.00 | 0.16 | 0.46 | 0.00 | 0.97 | 0.10 |
| TAI | 2014 | Differed | 0.10 | 0.53 | 0.25 | 0.17 | 0.21 | 0.49 | 0.17 | 0.00 | 0.49 | 0.35 | 0.00 | 0.76 | 0.10 |
| TAI | 2014 | Exclusion | 0.10 | 0.50 | 0.15 | 0.10 | 0.19 | 0.10 | 0.10 | 0.00 | 0.34 | 0.23 | 0.10 | 0.96 | 0.10 |
| APA | 2010 | Continuous | 0.10 | 1.10 | 0.10 | 0.10 | 0.10 | 0.30 | 0.16 | 0.00 | 0.12 | 0.78 | 0.00 | 0.85 | 0.10 |
| APA | 2010 | Differed | 0.10 | 0.55 | 0.10 | 0.10 | 0.11 | 0.65 | 0.13 | 0.10 | 0.15 | 0.41 | 0.00 | 0.85 | 0.10 |
| APA | 2010 | Exclusion | 0.10 | 0.00 | 0.14 | 0.10 | 0.10 | 0.73 | 0.10 | 0.33 | 0.14 | 0.25 | 0.00 | 1.08 | 0.10 |
| APA | 2011 | Continuous | 0.30 | 0.84 | 0.10 | 0.10 | 0.26 | 1.00 | 0.38 | 0.00 | 0.14 | 0.25 | 0.00 | 0.92 | 0.10 |
| APA | 2011 | Differed | 0.18 | 0.10 | 0.35 | 0.10 | 0.18 | 0.53 | 0.15 | 0.10 | 0.20 | 0.45 | 0.00 | 0.88 | 0.10 |
| APA | 2011 | Exclusion | 0.00 | 1.00 | 0.20 | 0.10 | 0.12 | 0.82 | 0.10 | 0.00 | 0.25 | 0.25 | 0.00 | 1.10 | 0.19 |
| APA | 2012 | Continuous | 0.25 | 0.68 | 0.15 | 0.10 | 0.29 | 0.26 | 0.32 | 0.00 | 0.16 | 0.22 | 0.00 | 0.99 | 0.10 |
| APA | 2012 | Differed | 0.30 | 0.10 | 0.42 | 0.10 | 0.16 | 0.20 | 0.26 | 0.10 | 0.12 | 0.34 | 0.00 | 0.83 | 0.10 |
| APA | 2012 | Exclusion | 0.00 | 0.30 | 0.15 | 0.10 | 0.13 | 0.43 | 0.30 | 0.00 | 0.21 | 0.18 | 0.00 | 0.81 | 0.10 |
| APA | 2013 | Continuous | 0.14 | 0.53 | 0.17 | 0.14 | 0.36 | 0.43 | 0.29 | 0.00 | 0.13 | 0.44 | 0.00 | 0.90 | 0.15 |
| APA | 2013 | Differed | 0.41 | 0.10 | 0.31 | 0.10 | 0.12 | 0.40 | 0.16 | 0.10 | 0.12 | 0.28 | 0.00 | 0.91 | 0.10 |
| APA | 2013 | Exclusion | 0.14 | 0.10 | 0.21 | 0.10 | 0.15 | 0.40 | 0.18 | 0.00 | 0.15 | 0.15 | 0.00 | 0.90 | 0.10 |
| APA | 2014 | Continuous | 0.13 | 1.00 | 0.10 | 0.10 | 0.15 | 0.37 | 0.18 | 0.00 | 0.13 | 0.45 | 0.00 | 0.74 | 0.10 |
| APA | 2014 | Differed | 0.15 | 0.10 | 0.16 | 0.10 | 0.15 | 0.18 | 0.10 | 0.10 | 0.12 | 0.31 | 0.00 | 0.89 | 0.10 |
| APA | 2014 | Exclusion | 0.10 | 0.55 | 0.10 | 0.10 | 0.16 | 0.10 | 0.16 | 0.00 | 0.13 | 0.10 | 0.00 | 0.88 | 0.10 |
