# Supplementary material for: Long-term ecological research in southern Brazil grasslands: Effects of grazing exclusion and deferred grazing on plant and arthropod communities
Source: PLoS One. 2020 Jan 13;15(1):e0227706. doi: 10.1371/journal.pone.0227706 (PMC6957338; doi:10.1371/journal.pone.0227706)
Supplement: S4 Table — Sites: ACE = Aceguá municipality, ALE = Alegrete municipality, ARA = Aratinga Ecological Station, LAV = Lavras do Sul municipality, TAI = Tainhas State Park, APA = Aparados da Serra National Park. Treatments refer to continuous or differed (intermittent) grazing, and grazing exclusion. (DOCX) [file pone.0227706.s004.docx]

| **Site** | **Year** | **Treatment** | **Araneae** | **Coleoptera** | **Diptera** | **Hemiptera** | **Hymenoptera** | **Orthoptera** | **Thysanoptera** |
| --- | --- | --- | --- | --- | --- | --- | --- | --- | --- |
| ACE | 2011 | Continuous grazing | 0 | 0 | 1 | 0 | 0 | 1 | 0 |
| ACE | 2011 | Differed grazing | 7 | 18 | 11 | 8 | 2 | 0 | 0 |
| ACE | 2011 | Grazing exclusion | 8 | 9 | 15 | 19 | 5 | 2 | 1 |
| ACE | 2012 | Continuous grazing | 80 | 9 | 63 | 114 | 112 | 20 | 17 |
| ACE | 2012 | Differed grazing | 58 | 33 | 387 | 150 | 94 | 23 | 1 |
| ACE | 2012 | Grazing exclusion | 176 | 115 | 101 | 337 | 205 | 19 | 51 |
| ACE | 2013 | Continuous grazing | 26 | 33 | 79 | 224 | 55 | 72 | 23 |
| ACE | 2013 | Differed grazing | 73 | 109 | 173 | 377 | 33 | 68 | 38 |
| ACE | 2013 | Grazing exclusion | 113 | 83 | 231 | 371 | 179 | 67 | 29 |
| ACE | 2014 | Continuous grazing | 9 | 6 | 12 | 85 | 32 | 15 | 0 |
| ACE | 2014 | Differed grazing | 39 | 14 | 61 | 66 | 15 | 23 | 0 |
| ACE | 2014 | Grazing exclusion | 18 | 12 | 15 | 24 | 16 | 1 | 1 |
| ALE | 2011 | Continuous grazing | 4 | 6 | 0 | 70 | 112 | 13 | 1 |
| ALE | 2011 | Differed grazing | 18 | 25 | 11 | 88 | 67 | 3 | 2 |
| ALE | 2011 | Grazing exclusion | 25 | 23 | 28 | 162 | 127 | 17 | 10 |
| ALE | 2012 | Continuous grazing | 36 | 36 | 52 | 224 | 884 | 72 | 7 |
| ALE | 2012 | Differed grazing | 55 | 27 | 36 | 135 | 511 | 40 | 19 |
| ALE | 2012 | Grazing exclusion | 96 | 95 | 279 | 511 | 575 | 61 | 141 |
| ALE | 2013 | Continuous grazing | 34 | 23 | 49 | 115 | 172 | 42 | 2 |
| ALE | 2013 | Differed grazing | 44 | 33 | 58 | 254 | 163 | 80 | 9 |
| ALE | 2013 | Grazing exclusion | 42 | 82 | 82 | 510 | 141 | 47 | 7 |
| ALE | 2014 | Continuous grazing | 9 | 12 | 26 | 66 | 95 | 13 | 0 |
| ALE | 2014 | Differed grazing | 27 | 31 | 82 | 232 | 165 | 43 | 25 |
| ALE | 2014 | Grazing exclusion | 39 | 48 | 81 | 210 | 62 | 41 | 15 |
| ARA | 2011 | Continuous grazing | 0 | 4 | 3 | 57 | 3 | 2 | 0 |
| ARA | 2011 | Differed grazing | 0 | 5 | 16 | 75 | 0 | 16 | 0 |
| ARA | 2011 | Grazing exclusion | 10 | 8 | 10 | 83 | 47 | 4 | 2 |
| ARA | 2012 | Continuous grazing | 15 | 43 | 23 | 57 | 16 | 14 | 0 |
| ARA | 2012 | Differed grazing | 11 | 15 | 17 | 24 | 4 | 16 | 2 |
| ARA | 2012 | Grazing exclusion | 32 | 8 | 18 | 63 | 25 | 14 | 0 |
| ARA | 2013 | Continuous grazing | 14 | 57 | 43 | 200 | 22 | 36 | 3 |
| ARA | 2013 | Differed grazing | 29 | 64 | 101 | 146 | 30 | 36 | 0 |
| ARA | 2013 | Grazing exclusion | 65 | 65 | 182 | 136 | 30 | 32 | 7 |
| ARA | 2014 | Continuous grazing | 21 | 31 | 46 | 156 | 36 | 36 | 0 |
| ARA | 2014 | Differed grazing | 40 | 31 | 97 | 163 | 26 | 42 | 3 |
| ARA | 2014 | Grazing exclusion | 31 | 14 | 30 | 32 | 12 | 13 | 1 |
| LAV | 2011 | Continuous grazing | 37 | 19 | 84 | 411 | 255 | 14 | 110 |
| LAV | 2011 | Differed grazing | 46 | 58 | 341 | 258 | 144 | 21 | 91 |
| LAV | 2011 | Grazing exclusion | 44 | 21 | 158 | 315 | 132 | 30 | 271 |
| LAV | 2012 | Continuous grazing | 78 | 19 | 130 | 308 | 340 | 17 | 10 |
| LAV | 2012 | Differed grazing | 109 | 102 | 385 | 594 | 455 | 90 | 244 |
| LAV | 2012 | Grazing exclusion | 106 | 30 | 89 | 464 | 183 | 125 | 62 |
| LAV | 2013 | Continuous grazing | 53 | 26 | 58 | 226 | 98 | 47 | 67 |
| LAV | 2013 | Differed grazing | 45 | 36 | 69 | 283 | 84 | 77 | 132 |
| LAV | 2013 | Grazing exclusion | 35 | 29 | 38 | 577 | 78 | 38 | 4 |
| LAV | 2014 | Continuous grazing | 0 | 1 | 47 | 8 | 9 | 3 | 0 |
| LAV | 2014 | Differed grazing | 29 | 22 | 92 | 180 | 53 | 17 | 1 |
| LAV | 2014 | Grazing exclusion | 31 | 58 | 34 | 1093 | 53 | 57 | 2 |
| TAI | 2011 | Continuous grazing | 31 | 1 | 23 | 63 | 5 | 9 | 11 |
| TAI | 2011 | Differed grazing | 40 | 12 | 34 | 48 | 10 | 24 | 8 |
| TAI | 2011 | Grazing exclusion | 31 | 14 | 27 | 62 | 10 | 14 | 11 |
| TAI | 2012 | Continuous grazing | 65 | 48 | 61 | 87 | 53 | 42 | 9 |
| TAI | 2012 | Differed grazing | 29 | 32 | 66 | 97 | 43 | 55 | 37 |
| TAI | 2012 | Grazing exclusion | 44 | 54 | 37 | 116 | 31 | 34 | 26 |
| TAI | 2013 | Continuous grazing | 37 | 22 | 115 | 177 | 42 | 36 | 19 |
| TAI | 2013 | Differed grazing | 41 | 47 | 109 | 166 | 48 | 44 | 20 |
| TAI | 2013 | Grazing exclusion | 74 | 80 | 179 | 192 | 38 | 44 | 71 |
| TAI | 2014 | Continuous grazing | 17 | 6 | 30 | 63 | 27 | 41 | 0 |
| TAI | 2014 | Differed grazing | 66 | 76 | 74 | 163 | 41 | 41 | 0 |
| TAI | 2014 | Grazing exclusion | 10 | 11 | 21 | 56 | 18 | 38 | 5 |
| APA | 2012 | Continuous grazing | 20 | 60 | 55 | 167 | 11 | 22 | 0 |
| APA | 2012 | Differed grazing | 12 | 45 | 40 | 70 | 9 | 10 | 0 |
| APA | 2012 | Grazing exclusion | 25 | 92 | 52 | 117 | 10 | 3 | 0 |
| APA | 2013 | Continuous grazing | 40 | 228 | 143 | 225 | 33 | 22 | 0 |
| APA | 2013 | Differed grazing | 21 | 85 | 177 | 234 | 19 | 17 | 3 |
| APA | 2013 | Grazing exclusion | 55 | 83 | 222 | 148 | 65 | 15 | 6 |
| APA | 2014 | Continuous grazing | 8 | 53 | 15 | 49 | 2 | 15 | 1 |
| APA | 2014 | Differed grazing | 8 | 28 | 37 | 65 | 7 | 12 | 0 |
| APA | 2014 | Grazing exclusion | 25 | 60 | 48 | 118 | 46 | 25 | 0 |
