# Supplementary material for: Long-term ecological research in southern Brazil grasslands: Effects of grazing exclusion and deferred grazing on plant and arthropod communities
Source: PLoS One. 2020 Jan 13;15(1):e0227706. doi: 10.1371/journal.pone.0227706 (PMC6957338; doi:10.1371/journal.pone.0227706)
Supplement: S5 Table — Sites: ACE = Aceguá municipality, ALE = Alegrete municipality, ARA = Aratinga Ecological Station, LAV = Lavras do Sul municipality, TAI = Tainhas State Park, APA = Aparados da Serra National Park. Treatments refer to continuous or differed (intermittent) grazing, and grazing exclusion. (DOCX) [file pone.0227706.s005.docx]

| **Site** | **Year** | **Treatment** | **Araneae** | **Coleoptera** | **Diptera** | **Hemiptera** | **Hymenoptera** | **Orthoptera** | **Thysanoptera** |
| --- | --- | --- | --- | --- | --- | --- | --- | --- | --- |
| ACE | 2011 | Continuous grazing | 82 | 39 | 118 | 28 | 281 | 7 | 11 |
| ACE | 2011 | Differed grazing | 235 | 52 | 791 | 245 | 288 | 2 | 60 |
| ACE | 2011 | Grazing exclusion | 382 | 46 | 1026 | 86 | 245 | 3 | 21 |
| ACE | 2012 | Continuous grazing | 95 | 60 | 100 | 84 | 623 | 4 | 45 |
| ACE | 2012 | Differed grazing | 124 | 185 | 167 | 56 | 675 | 23 | 2 |
| ACE | 2012 | Grazing exclusion | 151 | 103 | 335 | 168 | 453 | 15 | 21 |
| ACE | 2013 | Continuous grazing | 32 | 26 | 35 | 24 | 199 | 5 | 2 |
| ACE | 2013 | Differed grazing | 114 | 69 | 132 | 28 | 294 | 8 | 18 |
| ACE | 2013 | Grazing exclusion | 145 | 32 | 56 | 54 | 203 | 10 | 12 |
| ACE | 2014 | Continuous grazing | 43 | 22 | 25 | 1 | 231 | 11 | 3 |
| ACE | 2014 | Differed grazing | 97 | 31 | 42 | 10 | 112 | 6 | 0 |
| ACE | 2014 | Grazing exclusion | 70 | 30 | 25 | 17 | 292 | 4 | 2 |
| ALE | 2011 | Continuous grazing | 60 | 33 | 54 | 38 | 1109 | 5 | 39 |
| ALE | 2011 | Differed grazing | 80 | 20 | 37 | 19 | 763 | 13 | 14 |
| ALE | 2011 | Grazing exclusion | 72 | 25 | 26 | 44 | 420 | 15 | 3 |
| ALE | 2012 | Continuous grazing | 43 | 81 | 16 | 23 | 806 | 4 | 19 |
| ALE | 2012 | Differed grazing | 61 | 83 | 39 | 26 | 1179 | 3 | 15 |
| ALE | 2012 | Grazing exclusion | 116 | 108 | 45 | 16 | 559 | 15 | 51 |
| ALE | 2013 | Continuous grazing | 54 | 28 | 20 | 3 | 458 | 9 | 3 |
| ALE | 2013 | Differed grazing | 94 | 42 | 34 | 22 | 410 | 7 | 22 |
| ALE | 2013 | Grazing exclusion | 65 | 29 | 17 | 26 | 202 | 3 | 0 |
| ALE | 2014 | Continuous grazing | 26 | 24 | 12 | 9 | 224 | 12 | 2 |
| ALE | 2014 | Differed grazing | 73 | 68 | 42 | 32 | 267 | 0 | 1 |
| ALE | 2014 | Grazing exclusion | 99 | 73 | 66 | 8 | 273 | 5 | 2 |
| ARA | 2011 | Continuous grazing | 64 | 33 | 71 | 55 | 118 | 4 | 101 |
| ARA | 2011 | Differed grazing | 42 | 19 | 33 | 47 | 53 | 12 | 11 |
| ARA | 2011 | Grazing exclusion | 68 | 41 | 60 | 100 | 155 | 25 | 6 |
| ARA | 2012 | Continuous grazing | 176 | 103 | 68 | 21 | 633 | 10 | 31 |
| ARA | 2012 | Differed grazing | 36 | 63 | 194 | 36 | 331 | 11 | 6 |
| ARA | 2012 | Grazing exclusion | 11 | 48 | 131 | 30 | 171 | 75 | 3 |
| ARA | 2013 | Continuous grazing | 47 | 107 | 10 | 10 | 656 | 8 | 3 |
| ARA | 2013 | Differed grazing | 29 | 32 | 7 | 26 | 364 | 14 | 1 |
| ARA | 2013 | Grazing exclusion | 10 | 19 | 12 | 6 | 98 | 5 | 1 |
| ARA | 2014 | Continuous grazing | 68 | 226 | 9 | 28 | 457 | 0 | 0 |
| ARA | 2014 | Differed grazing | 43 | 116 | 9 | 32 | 237 | 3 | 6 |
| ARA | 2014 | Grazing exclusion | 9 | 22 | 14 | 6 | 106 | 2 | 0 |
| LAV | 2011 | Continuous grazing | 88 | 151 | 69 | 28 | 1026 | 16 | 27 |
| LAV | 2011 | Differed grazing | 150 | 317 | 178 | 41 | 818 | 22 | 23 |
| LAV | 2011 | Grazing exclusion | 85 | 212 | 81 | 18 | 455 | 2 | 64 |
| LAV | 2012 | Continuous grazing | 109 | 139 | 195 | 67 | 963 | 7 | 101 |
| LAV | 2012 | Differed grazing | 111 | 284 | 350 | 49 | 1557 | 7 | 91 |
| LAV | 2012 | Grazing exclusion | 88 | 209 | 94 | 37 | 680 | 3 | 19 |
| LAV | 2013 | Continuous grazing | 82 | 306 | 52 | 1 | 636 | 30 | 1 |
| LAV | 2013 | Differed grazing | 108 | 237 | 79 | 9 | 485 | 24 | 1 |
| LAV | 2013 | Grazing exclusion | 24 | 122 | 79 | 9 | 322 | 2 | 0 |
| LAV | 2014 | Continuous grazing | 36 | 136 | 11 | 6 | 269 | 54 | 1 |
| LAV | 2014 | Differed grazing | 44 | 177 | 60 | 8 | 547 | 16 | 4 |
| LAV | 2014 | Grazing exclusion | 43 | 123 | 25 | 13 | 181 | 4 | 4 |
| TAI | 2011 | Continuous grazing | 113 | 24 | 15 | 55 | 235 | 22 | 55 |
| TAI | 2011 | Differed grazing | 50 | 24 | 20 | 26 | 155 | 22 | 17 |
| TAI | 2011 | Grazing exclusion | 57 | 22 | 12 | 35 | 176 | 20 | 11 |
| TAI | 2012 | Continuous grazing | 44 | 90 | 23 | 10 | 290 | 18 | 72 |
| TAI | 2012 | Differed grazing | 30 | 59 | 33 | 6 | 128 | 24 | 74 |
| TAI | 2012 | Grazing exclusion | 32 | 50 | 14 | 8 | 91 | 43 | 39 |
| TAI | 2013 | Continuous grazing | 53 | 22 | 39 | 21 | 90 | 16 | 4 |
| TAI | 2013 | Differed grazing | 22 | 47 | 46 | 31 | 81 | 13 | 31 |
| TAI | 2013 | Grazing exclusion | 63 | 45 | 22 | 20 | 63 | 7 | 1 |
| TAI | 2014 | Continuous grazing | 18 | 40 | 91 | 5 | 159 | 2 | 1 |
| TAI | 2014 | Differed grazing | 30 | 67 | 21 | 22 | 74 | 4 | 12 |
| TAI | 2014 | Grazing exclusion | 31 | 20 | 24 | 7 | 71 | 7 | 0 |
| APA | 2012 | Continuous grazing | 159 | 125 | 131 | 22 | 278 | 5 | 26 |
| APA | 2012 | Differed grazing | 39 | 98 | 116 | 33 | 263 | 16 | 25 |
| APA | 2012 | Grazing exclusion | 122 | 90 | 49 | 17 | 911 | 22 | 4 |
| APA | 2013 | Continuous grazing | 57 | 64 | 17 | 39 | 142 | 2 | 4 |
| APA | 2013 | Differed grazing | 64 | 41 | 12 | 59 | 120 | 2 | 7 |
| APA | 2013 | Grazing exclusion | 24 | 27 | 20 | 30 | 293 | 7 | 1 |
| APA | 2014 | Continuous grazing | 173 | 30 | 7 | 6 | 86 | 5 | 2 |
| APA | 2014 | Differed grazing | 64 | 29 | 22 | 18 | 59 | 0 | 1 |
| APA | 2014 | Grazing exclusion | 39 | 25 | 20 | 46 | 176 | 2 | 0 |
