# Supplementary figures and images for: Long-term ecological research in southern Brazil grasslands: Effects of grazing exclusion and deferred grazing on plant and arthropod communities
Source: PLoS One. 2020 Jan 13;15(1):e0227706. doi: 10.1371/journal.pone.0227706 (PMC6957338; doi:10.1371/journal.pone.0227706)

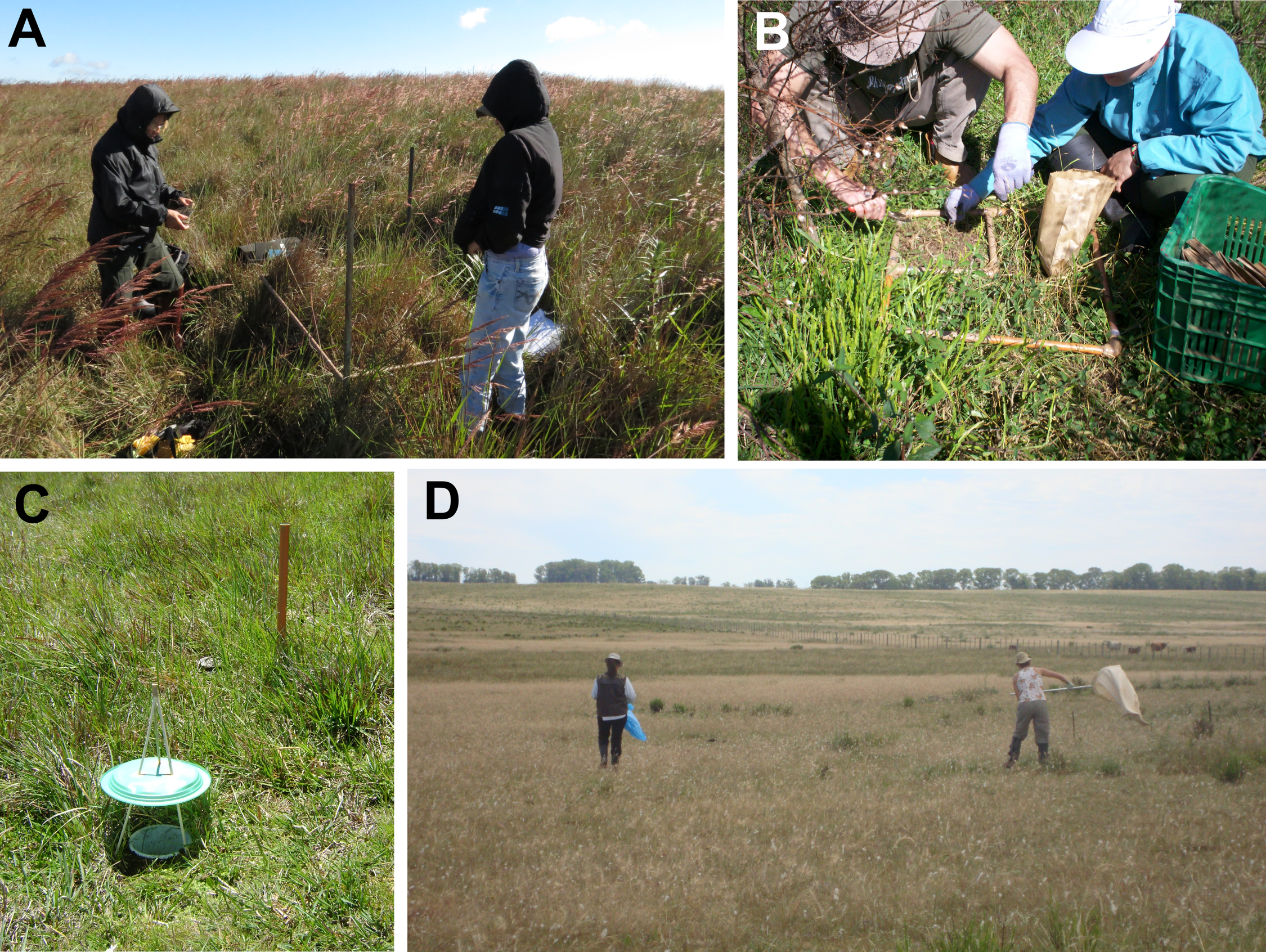

Supplement: S1 Fig — A. Plant community survey in 1m2 subplots (alpha component; n = 162). B. Biomass sampling. C. Pitfall trap used to sample epigeic arthropods. D. Sweeping nets used to sample vegetation arthropods. (TIF) [file pone.0227706.s006.tif]
